# Dynamics of visual object coding within and across the hemispheres: Objects in the periphery

**DOI:** 10.1126/sciadv.adq0889

**Published:** 2025-01-01

**Authors:** Amanda K. Robinson, Tijl Grootswagers, Sophia M. Shatek, Marlene Behrmann, Thomas A. Carlson

**Affiliations:** ^1^ School of Psychology, The University of Queensland, Brisbane, Australia.; ^2^ Queensland Brain Institute, The University of Queensland, Brisbane, Australia.; ^3^ School of Psychology, University of Sydney, Sydney, Australia.; ^4^ The MARCS Institute for Brain, Behaviour and Development, Western Sydney University, Sydney, Australia.; ^5^ School of Computer, Data and Mathematical Sciences, Western Sydney University, Sydney, Australia.; ^6^ Department of Experimental Psychology, University of Oxford, Oxford, UK.; ^7^ Department of Psychology, Carnegie Mellon University, Pittsburgh, PA 15213, USA.; ^8^ Department of Ophthalmology, University of Pittsburgh, Pittsburgh, PA 15260, USA.

## Abstract

The human brain continuously integrates information across its two hemispheres to construct a coherent representation of the perceptual world. Characterizing how visual information is represented in each hemisphere over time is crucial for understanding how hemispheric transfer contributes to perception. Here, we investigated information processing within each hemisphere over time and the degree to which it is distinct or duplicated across hemispheres. We presented participants with object images lateralized to the left or right visual fields while measuring their brain activity with electroencephalography. Stimulus coding was more robust and emerged earlier in the contralateral than the ipsilateral hemisphere. Presentation of two stimuli, one to each hemifield, reduced the fidelity of representations in both hemispheres relative to one stimulus alone, signifying hemispheric interference. Last, we found that processing within the contralateral, but not ipsilateral, hemisphere was biased to image-related over concept-related information. Together, these results suggest that hemispheric transfer operates to filter irrelevant information and efficiently prioritize processing of meaning.

## Teaser

A sequence of neural computations underpins the integration of visuoperceptual information across the two hemispheres of the brain.

## INTRODUCTION

The human brain has two distinct but connected hemispheres that must communicate and coordinate to yield unitary visual perception. Because of the contralateral arrangement of the visual system, stimuli presented to one hemifield are initially processed in the opposite hemisphere. Yet, from this hemisphere-distinct processing, a single coherent percept of the visual world emerges, highlighting the importance of hemispheric integration. The nature of hemispheric processing has fascinated cognitive neuroscientists for decades. For example, studies using split-brain patients, who had the corpus callosum surgically severed, have shown that the left and right hemispheres can have different perceptual experiences and responses to the same stimulus (*1*, *2*). The mechanisms underlying hemispheric transfer are complex, involving communication between brain regions at different levels of processing, but understanding how neural processing results in perception requires a better understanding of information processing within and across hemispheres.

The left and right hemispheres of the brain are largely homologous in terms of structure and function. The strength in this duplication, or redundancy, can be borne out in behavior; for example, interhemispheric cooperation can improve performance on highly complex tasks (*3*, *4*). Neurally, interhemispheric communication is facilitated by a number of anatomical connections between the left and right hemispheres, including the corpus callosum, posterior commissure, and anterior commissure (*5*–*7*). These connections allow the two hemispheres to share information and coordinate their activities. For visual perception, the integration of information across the hemispheres is crucial yet is not as well characterized as other aspects of visual processing. The hierarchical nature of visual processing has been well-studied; across the swath of visual cortex, there is a hierarchical flow of features, starting with sensitivity to low-level features such as straight edges coded in primary visual cortex, through successive stages to higher-level categorical features associated with specific patterns (e.g., words or faces) coded in ventral temporal cortex (*8*, *9*). Models of the visual system, however, typically do not consider the interplay between the two hemispheres, despite their joint involvement in perception. Here, we map the dynamics of information coding within each hemisphere and the sharing of this information across hemispheres.

Neural recordings have shed light on the computations that underlie hemispheric processing. Studies using electroencephalography (EEG) in humans have shown earlier and stronger evoked responses over the scalp contralateral to stimulus presentation relative to the ipsilateral side, consistent with the trajectory of fibers from the eyes to the contralateral hemisphere (*10*–*12*). Interhemispheric transfer time calculated from event-related potentials in occipital brain regions have been observed between 13 and 26 ms (*10*, *12*, *13*), varying by stimulus location and intensity (*14*, *15*). Other work has used functional magnetic resonance imaging to characterize retinotopic biases within the brain, specifically showing the dominance of contralateral responses for visual processing throughout occipital cortex (*16*) and parietal cortex (*17*, *18*), as well as other regions of the brain such as the hippocampus (*19*). Regions of visual cortex typically considered category-selective exhibit contralateral biases and distinct responses across the hemispheres (*20*, *21*). This vast coding of visuospatial maps with contralateral biases within the brain has implications for cognitive functioning (*22*). The inferred but untested implication is that the contralateral hemisphere initially registers the sensory information and then propagates a subset of this information to the ipsilateral hemisphere. Yet, strength of neural activation is not necessarily correlated with strength of information coding (*23*). A pertinent question, then, and the focus of this paper is how the representations derived in the contralateral and ipsilateral hemispheres differ.

Studying hemispheric information has proven difficult in humans due to traditional analytic methods of neural data that obscure subtle neural patterns of activity. With the advent of multivariate pattern analyses (MVPA), or neural “decoding,” however, we can study what is represented in the brain using noninvasive neural recordings in humans (*24*). Combined with high temporal resolution neuroimaging methods such as EEG, MVPA can elucidate the time course and fidelity of stimulus information within neural patterns of activity. Time-resolved neural decoding methods have shown that visual information is represented quickly in the brain, occurring in less than 100 ms from stimulus onset (*25*, *26*). Notwithstanding this rapid time course, EEG has sufficient resolution to detect high spatial frequency neural activity early in the course of signal propagation (*27*), making it possible to separate signals from the left and right hemispheres and permitting opportunities to study dynamics of information within each hemisphere.

**Fig. 1.**
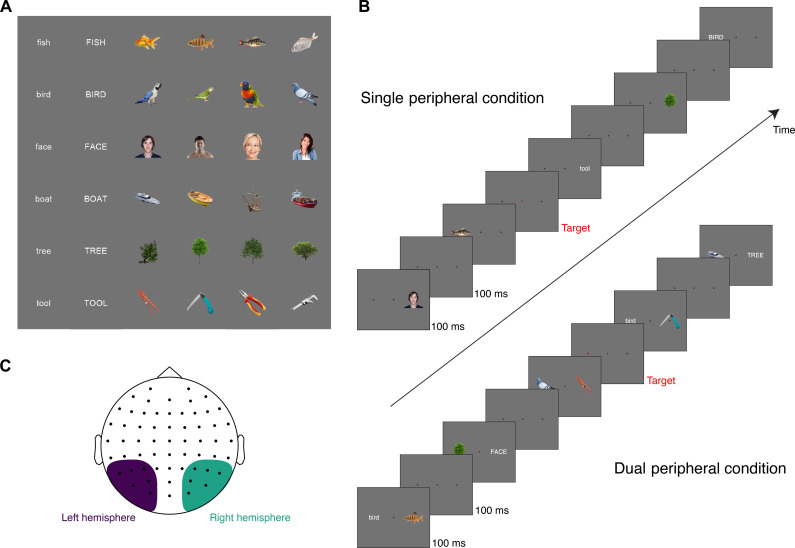
Experimental design. (**A**) Stimuli were 36 images of objects and matching word labels for six different object categories. (**B**) Example sequence timeline for single peripheral and dual peripheral conditions. (**C**) Electrode clusters for left hemisphere and right hemisphere analyses.

Here, we use EEG and multivariate analyses to investigate the representation of visual information within each hemisphere and the similarities across hemispheres over time. We used a rapid serial visual presentation paradigm with visual stimuli presented to the left and right hemifields, while neural activity was measured using EEG (Fig. 1). The goals of this study were threefold. First, we assessed how the contralateral and ipsilateral hemispheres process visual signals from the periphery (projected just to one hemisphere initially) over time. Second, we presented stimuli to the left and right hemifields simultaneously to understand how processing changes when different signals project to the two hemispheres, with the expectation that there might be interference in the representations. Last, to understand the content of hemispheric information, we assessed how the neural representations per hemisphere compare to similarity judgments on independent perceptual and conceptual tasks. We found clear contralateral dominance in the strength of visual coding, as expected. Further, we found reduction in information when two different stimuli were shown, one to each hemifield, suggesting that there may be interference or competition for representation between contralateral and (propagated) ipsilateral information. With respect to behavior, we found that the contralateral hemisphere contains more information relevant to perceptual than conceptual judgments but that this perceptual bias is largely missing in the ipsilateral hemisphere. This finding provocatively indicates that interhemispheric transfer might efficiently prioritize meaning rather than image statistics. These results yield great insights into hemispheric processing, the complex interplay between the two hemispheres, and how they cooperate to create a unified representation of the visual world.

## RESULTS

### Neural dynamics of stimulus representations: Single peripheral condition

To investigate hemispheric dynamics when processing was biased to one hemisphere, we assessed the neural representations of stimuli shown peripherally to the contralateral and ipsilateral visual fields (Fig. 1B, top). MVPA, or neural decoding, was applied to the time-resolved EEG data to discriminate how different stimuli evoked different patterns of neural activity over the scalp (*24*, *28*). For each presentation condition (single peripheral and dual peripheral), stimulus hemifield (left/right), hemisphere (left/right), time point, and participant, we assessed stimulus-specific representations by training a classifier to discriminate between neural activity associated with two experimental stimuli and testing on held out data for the same stimuli. This procedure was repeated for all pairs of the 36 experimental stimuli (630 stimulus pairs), and the mean pairwise decoding accuracy was assessed for the participant group per condition. Above-chance decoding accuracy (above 50%) was considered evidence of stimulus information in the neural signals.

**Fig. 2.**
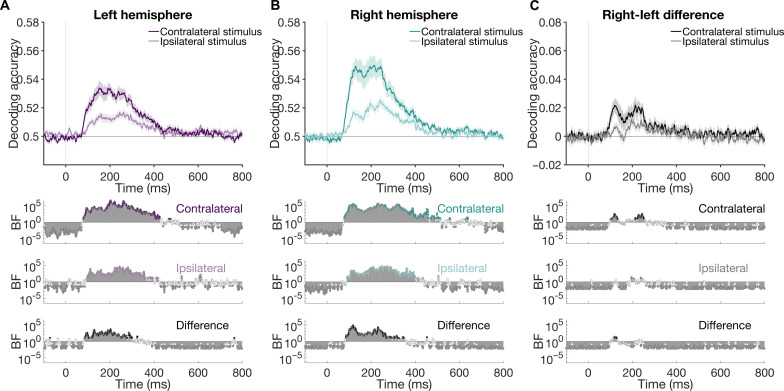
Peripheral stimulus representations in the left and right hemispheres (single peripheral condition). Neural representations for stimuli in the single peripheral condition across time (1-ms resolution), as indexed by mean decoding accuracy of pairs of experimental stimuli. Accuracy of decoding is plotted separately for stimuli appearing in the contralateral and ipsilateral visual fields. (**A**) Decoding using cluster of electrodes over the left hemisphere. (**B**) Decoding using cluster of electrodes over the right hemisphere. (**C**) Difference between right and left hemisphere decoding accuracy. Shaded lines indicate SEM. Contralateral representations were stronger, with earlier peaks and onsets than ipsilateral representations. Right hemisphere representations were stronger than the left for contralateral stimulus presentations. Bottom plots show Bayes factors (BFs) which indicate the evidence for above-chance decoding per condition or nonzero differences between conditions.

Decoding was performed separately using clusters of electrodes measuring from the left and right hemispheres over time (Fig. 1C). Stimulus information coded within each hemisphere, as indexed by mean pairwise decoding accuracy, showed clear contralateral dominance (Fig. 2, A and B). In the left hemisphere, right visual field (RVF) stimuli showed higher decoding and earlier onset (peak = 53.39%, onset = 79 ms) than left visual field (LVF) stimuli (peak = 51.68%, onset = 96 ms). Similarly, in the right hemisphere, the contralateral (LVF) stimuli showed higher decoding and earlier onsets (peak = 54.98%, onset = 78 ms) than RVF stimuli (peak = 52.53%, onset = 91 ms). Furthermore, comparison of peak times for each hemifield revealed earlier peak decoding for the contralateral hemisphere than the ipsilateral hemisphere (see Table 1). Representations in the right hemisphere were stronger than those in the left hemisphere for both contralateral and ipsilateral stimuli (Fig. 2C), consistent with previous reports (*29*). Notably, this right-hemispheric dominance was not driven by the faces in the stimulus set; the 18 nonface objects also produced stronger right hemisphere representations when analyzed separately (see the Supplementary Materials). Overall, decoding of single stimuli presented to a hemifield provided support for differing dynamics in each hemisphere and a contralateral precedence in the visual system. Next, we turned to how the dynamics of representation varied when there were competing visual inputs from the two hemifields.

**Table 1.** Onset time (with 95% confidence intervals in brackets), time of peak decoding, and peak decoding accuracy for single and dual peripheral conditions. Onsets and peaks according to condition (single/dual peripheral), hemisphere, and side of stimulus presentation (LVF/RVF). Contralateral combinations are in bold. Onsets were calculated as the time of the first time Bayes factors were above 10 for 10 ms consecutively. Confidence intervals were calculated using jackknifing to subsample participants (leave-two-participants-out; 190 unique permutations) to yield a distribution of onset and peak times.

		Onset time		Time of peak decoding		Peak decoding accuracy	
		LH	RH	LH	RH	LH	RH
Single peripheral	LVF	96 [91–104]	**78 [77–79]**	244 [244–262]	**214 [129–243]**	51.68%	**54.98%**
	RVF	**79 [77–81]**	91 [90–104]	**152 [152–201]**	241 [241–241]	**53.39%**	52.53%
Dual peripheral	LVF	99 [97–105]	**78 [76–82]**	254 [111–254]	**115 [114–215]**	50.93%	**54.49%**
	RVF	**69 [68–78]**	85 [79–87]	**202 [202–204]**	245 [244–257]	**53.13%**	51.27%

### Neural dynamics of stimulus representations: Dual peripheral condition

**Fig. 3.**
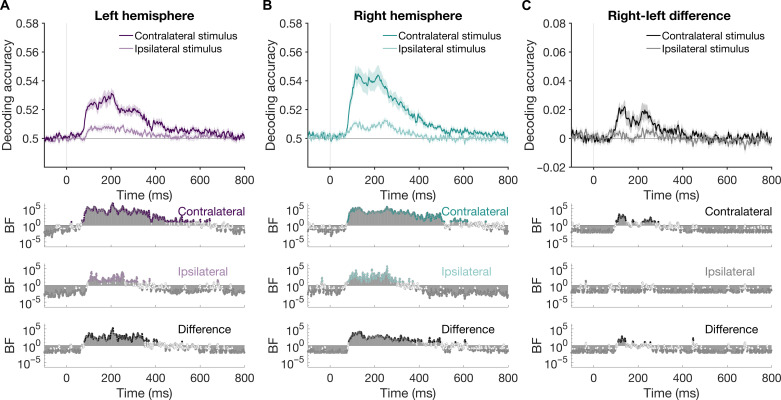
Peripheral stimulus representations in the left and right hemispheres (dual peripheral condition). Neural representations when two stimuli are presented to different hemispheres, as indexed by mean pairwise decoding accuracy. Accuracy is plotted separately for stimuli appearing in the contralateral and ipsilateral visual fields. (**A**) Decoding using cluster of electrodes over the left hemisphere. (**B**) Decoding using cluster of electrodes over the right hemisphere. (**C**) Difference between right and left hemisphere decoding. Shaded lines represent SEM. Representations for the contralateral stimuli were stronger, with earlier peaks and onsets than for ipsilateral stimuli. Right hemisphere representations were stronger than the left for contralateral stimuli. Bottom plots show BFs which indicate the evidence for above-chance decoding per condition or nonzero differences between conditions.

We were interested in how representational dynamics varied according to the contralateral and ipsilateral sides when two stimuli were presented simultaneously: one to the LVF and one to the RVF (Fig. 3). Given the ubiquitous nature of hemispheric transfer, we expected that the fidelity of stimulus information might be reduced when there are competing inputs to the two hemispheres. For this dual peripheral condition, decoding was performed for each stimulus separately but, now, in the context of a second stimulus in the other field. Again, as above, there was clear contralateral dominance in both the left and right hemispheres (Fig. 3, A and B). In the left hemisphere, the RVF stimuli showed higher decoding and earlier onset (peak = 53.13%, onset = 69 ms) than LVF stimuli (peak = 50.93%, onset = 99 ms). Echoing these results, in the right hemisphere, the contralateral (LVF) stimuli showed higher decoding and earlier onsets (peak = 54.49%, onset = 78 ms) than RVF stimuli (peak = 51.27%, onset = 85 ms). Right hemisphere superiority was reliable for contralateral but not ipsilateral representations (Fig. 3C). Together, these results show that even when there is competing information from the two hemifields, information about each stimulus is processed by both hemispheres of the brain at the same time. Whether these dynamics were similar between the single and dual peripheral conditions was tested next.

### Comparison of single and dual peripheral presentation

To assess whether the addition of a second stimulus in the dual peripheral condition interfered with the fidelity of stimulus representations, we compared the timing and strength of decoding in the single and dual peripheral conditions. Inspection of decoding onset time revealed earlier onsets for contralateral than ipsilateral stimuli, with contralateral decoding beginning before 80 ms and ipsilateral decoding tending to begin after 90 ms, regardless of which hemisphere or condition was being assessed (Table 1). Thus, the initial stage of stimulus processing was not influenced by clutter in the display.

**Fig. 4.**
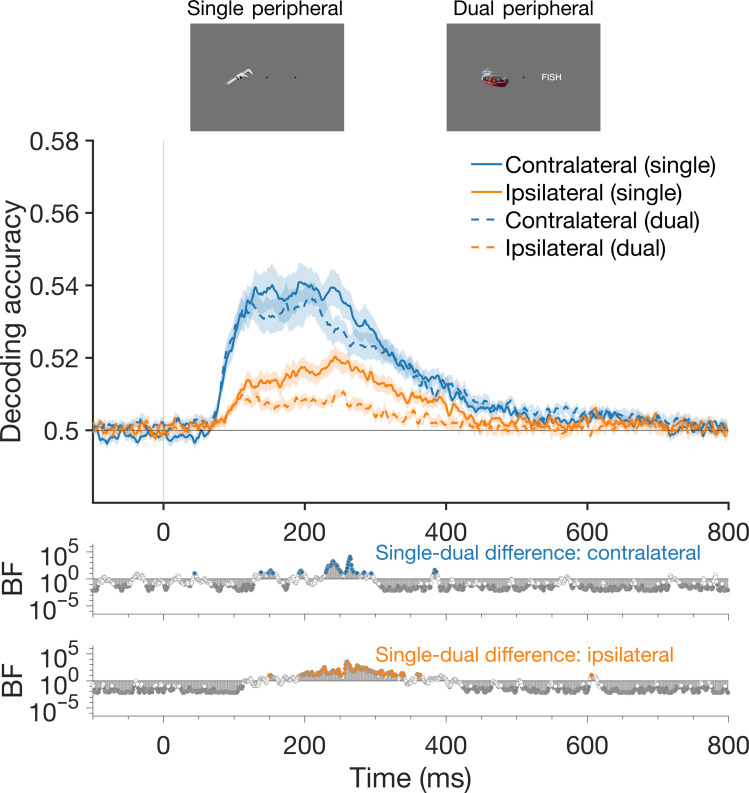
Decoding accuracy for peripheral stimuli when presented alone (single) or with another stimulus (dual). Decoding accuracy is plotted according to whether stimuli were contralateral or ipsilateral relative to the electrode cluster. These results are collapsed across the left and right hemispheres. Information was stronger in the single peripheral condition than the dual peripheral condition (see BFs for difference), for both contralateral and ipsilateral stimuli. Shaded lines represent SEM. Bottom plots show BFs indicating the evidence for nonzero differences between single and dual conditions.

However, there was a decrease in stimulus-related information in the brain in the dual peripheral relative to the single peripheral condition (Fig. 4). To compare the representations encoded by the brain between conditions, we assessed decoding accuracy in each condition for contralateral and ipsilateral stimuli, collapsed across the left and right hemispheres. For both contralateral and ipsilateral combinations, stimulus representations were more robust for the single peripheral than the dual peripheral condition, indicating that a stimulus presented in the other visual field decreased the fidelity of stimulus representations.

### Representational similarity analyses: Comparison across hemispheres

We were next interested in the structure of representations in the two hemispheres of the brain. The hypothesis was that stimulus coding in the ipsilateral hemisphere should reflect the same representations as the contralateral hemisphere but following a delay approximating interhemispheric transfer time. To investigate the relationship between representations in the two hemispheres, we used representational similarity analyses (RSA) (*30*), which abstracts away from specific activity patterns (e.g., from EEG electrodes over the left hemisphere) to the relationships between different stimulus representations.

**Fig. 5.**
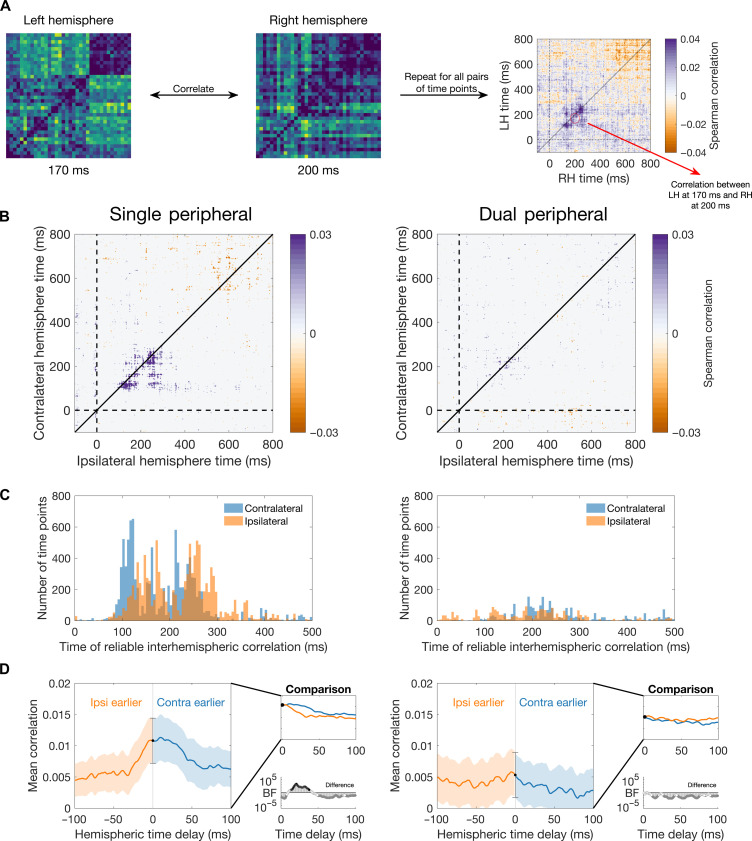
Shared structure of representations across the hemispheres. (**A**) Interhemispheric representational similarity approach for assessing shared information across the hemispheres. The 36 × 36 stimulus neural RDMs (an indexed by decoding accuracy) were correlated between the two hemispheres for every pair of time points using split-half cross-validation. This resulted in time × time hemispheric correlation matrices. If there was no delay between the hemispheres, then the time generalization plots would be symmetrical around the diagonal. (**B**) Plots show mean time × time correlations for representational structure in the contralateral and ipsilateral hemispheres for the single peripheral (left) and dual peripheral (right) conditions. Plots are thresholded by points with evidence for cross-hemispheric correlations different from zero (BF > 3). The highest correlations were observed for contralateral to ipsilateral delays in the peripheral conditions (i.e., below diagonal correlations). (**C**) Time of reliable interhemispheric correlations. Histograms show the number of reliable (BF > 3) positive hemispheric correlation time points as a function of contralateral and ipsilateral time. (**D**) Mean interhemispheric correlation values across participants for different ipsilateral-contralateral delays. Inset plot shows overlaid correlations as a function of absolute hemispheric time delay, and the plot below shows BFs associated with the differences between positive and negative delays. Correlations were higher for the positive contralateral earlier delays than the negative delays for the single peripheral but not the dual peripheral condition. RH, right hemisphere; LH, left hemisphere.

For each presentation condition (single peripheral and dual peripheral), stimulus visual field (LVF/RVF), time point, and participant, we constructed neural representational dissimilarity matrices (RDMs) to quantify the dissimilarity in the representations of all 36 stimuli (i.e., in a 36 × 36 matrix with 630 unique values). The RDMs from the left and right hemispheres were then correlated for all possible time points (Fig. 5A). These analyses were performed in a split-half manner, where correlations were always calculated across separate experimental sequences. Correlations between contralateral and ipsilateral hemispheres were assessed by collapsing across relevant visual field and left/right hemispheres. We found evidence for shared representations across the contralateral and ipsilateral hemispheres, particularly between 100 and 300 ms after stimulus onset (Fig. 5B). These correlations were more reliable for the single peripheral condition than the dual peripheral condition. This suggests that information that is transferred from the contralateral to ipsilateral hemisphere maintains a stimulus-specific structure but that transfer is reduced in the case of competing information (as in the dual peripheral condition).

To assess the timing of information sharing, we assessed the number of time points with reliable correlations [Bayes factor (BF) > 3] as a function of time for the contralateral and ipsilateral hemispheres. As expected, times were earlier for the contralateral than ipsilateral hemisphere (Fig. 5C). The time delay in hemispheric processing was assessed by calculating mean correlation values for a range of ipsilateral-contralateral delays per participant (−100 to 100 ms). These are the mean values for off-center diagonals between 0 and 500 ms of the time generalization RSA in Fig. 5B; negative time delays reflect diagonals shifted upward (ipsilateral earlier than contralateral), and positive delays reflect diagonals shifted downward (contralateral earlier than ipsilateral). In the single peripheral condition, the mean correlation was reliably higher for positive “contralateral earlier” than negative “ipsilateral earlier” offsets between 14 and 38 ms (BFs > 10). This provides support for an asymmetry in hemispheric processing, with contralateral processing preceding ipsilateral processing. The dual peripheral condition did not show a reliable asymmetry (Fig. 5D). The same pattern of results is observed when the analyses are restricted to the 24 object stimuli (see the Supplementary Materials).

### Consistency within and across hemispheres

**Fig. 6.**
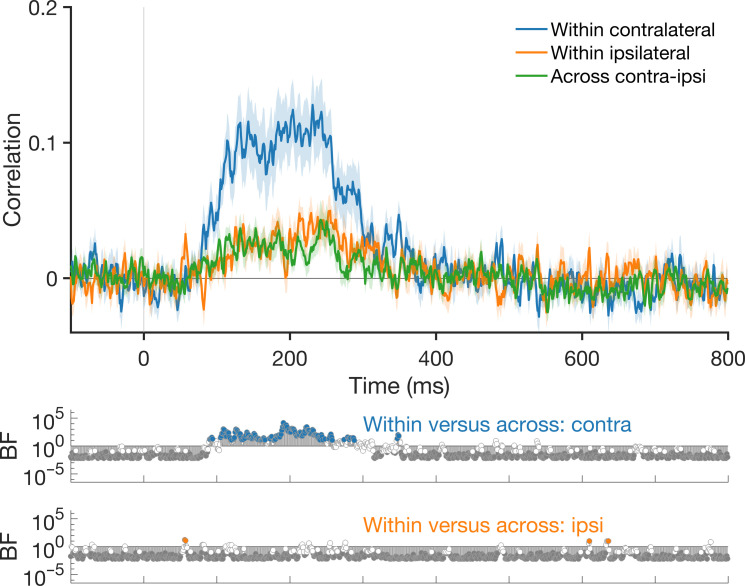
Consistency of information within versus across hemispheres. Correlations between representational structure within and across hemispheres show unique information in the contralateral hemisphere above information that is shared with the ipsilateral hemisphere but no unique information in the ipsilateral hemisphere. “Within” hemisphere consistency is calculated from a given hemisphere (contralateral/ipsilateral) using split-half Spearman correlation. “Across” hemisphere consistency is calculated as the correlation between the left and right hemispheres (as in Fig. 5). Results are shown from the single peripheral condition.

Next, we assessed the consistency in the structure of information within a hemisphere compared to information that is shared across hemispheres, to quantify unique versus shared hemispheric information. Within-hemisphere consistency was calculated as the correlation between RDMs from odd and even trial sequences for a given hemisphere. This correlation is a measure of reliability, which acts as a noise ceiling, the maximal correlation that could be expected with that hemisphere. Across-hemisphere consistency was calculated as the correlation between RDMs from the left and right hemispheres for odd and even trial sequences (as in Fig. 5). Mean within versus across hemisphere consistency for the single peripheral condition is shown in Fig. 6, collapsed across the left and right hemispheres to assess information within the contralateral and ipsilateral hemispheres relative to the shared information between them. Overall, there were higher correlations within the contralateral hemisphere than the shared (“across”) hemisphere correlations but no reliable differences between the ipsilateral consistency and the shared information. Together, these results suggest that the contralateral hemisphere contains unique information over and above that in the ipsilateral hemisphere but that the information in the ipsilateral hemisphere is a subset of that from the contralateral hemisphere.

### Behavioral relevance of neural representations

**Fig. 7.**
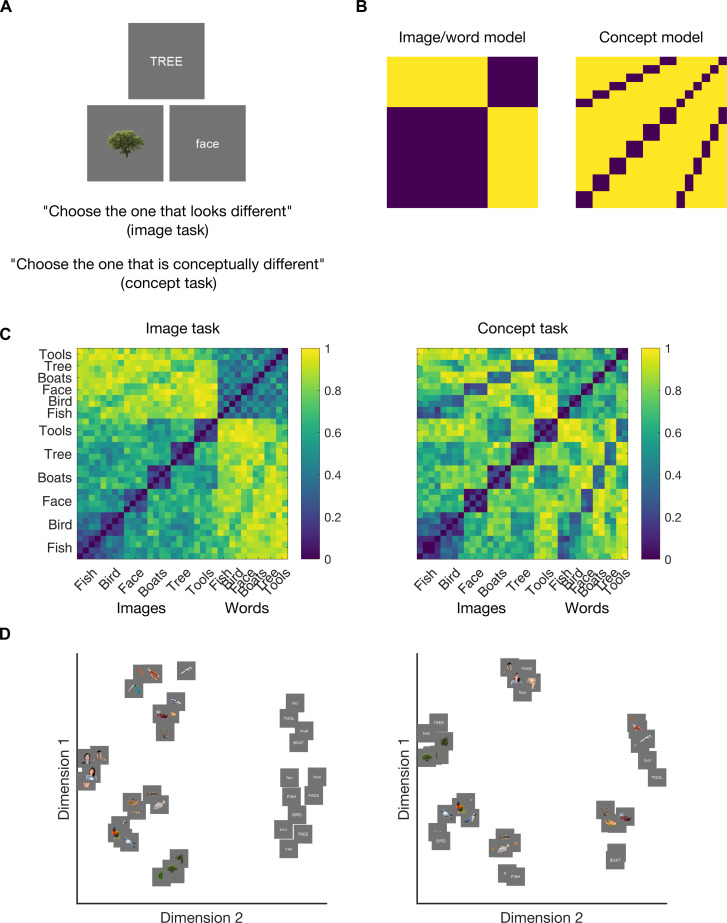
Behavioral similarity tasks and results. (**A**) Behavioral task design. Three stimuli were presented simultaneously, and the task was to “choose the image that looks different” (image task) or “choose the image that is conceptually different” (concept task). (**B**) Stimulus models constructed for the 36 stimuli based on whether stimuli were images or words (left) or shared the same concepts (right). (**C**) Dissimilarity matrices based on the results from each behavioral task show relationships between the stimuli based on image/word classification (left; image similarity task) and object type (right; concept similarity task). (**D**) Multidimensional scaling shows how stimuli clustered in the behavioral data. For the image similarity task (left), words formed a separate cluster from the images. For the conceptual similarity task (right), words and images of the same concept clustered together.

Having established that the representations of visual stimuli vary according to stimulus visual field and hemisphere and that these representations are shared across hemispheres, we assessed their behavioral relevance to give insight into the content of the neural representations. We collated the perceptual dissimilarity of the 36 experimental stimuli obtained using online experiments. In each experiment, participants were presented with three stimuli simultaneously and asked to choose the odd-one-out according to two sets of instructions: “choose the one that looks different” (image task) or “choose the one that is conceptually different” (concept task) (Fig. 7A). Stimulus dissimilarity was calculated as the proportion of odd-one-out choices for pairs of stimuli when they were presented together.

The behavioral results revealed that similarity judgments reflected both object category and meaning. To assess how the stimuli were clustered on the basis of similarity judgments per task, we correlated the behavioral RDMs (Fig. 7C) with models based on image/word category regardless of the object meaning (image model) and object type (concept model) (Fig. 7B) using Spearman correlation. The image model correlated with behavioral judgments in the image task (*r* = 0.81, *P* < 0.001) and the concept task (*r* = 0.28, *P* < 0.001). In addition, the concept model which distinguished the six object categories (bird, fish, tree, boat, face, and tools), regardless of image/word status, also correlated with behavior on the image task (*r* = 0.30, *P* < 0.001) and the concept task (*r* = 0.59, *P* < 0.001). The two behavioral RDMs were significantly correlated (*r* = 0.61, *P* < 0.001; see the Supplementary Materials for all model correlations). However, the image model had a higher correlation with the image similarity task than the concept similarity task (*z* = 20.96, *P* < 0.001), and the concept model had a lower correlation with behavior on the image task than the concept task (*z* = −9.68, *P* < 0.001). Thus, although both the image and concept similarity tasks are correlated with both the image and concept models and each other, there is a weighted asymmetry: Images and words were chosen as more dissimilar in the image similarity task than the concept task because they look different despite sharing meaning and were intermixed and more related to the concept of the stimulus independent of perceptual format in the concept task. This asymmetry can be seen in the multidimensional visualization of the results, where in the image task, words clustered separately to different image categories, but in the conceptual task, words clustered with their associated objects (Fig. 7, C and D).

**Fig. 8.**
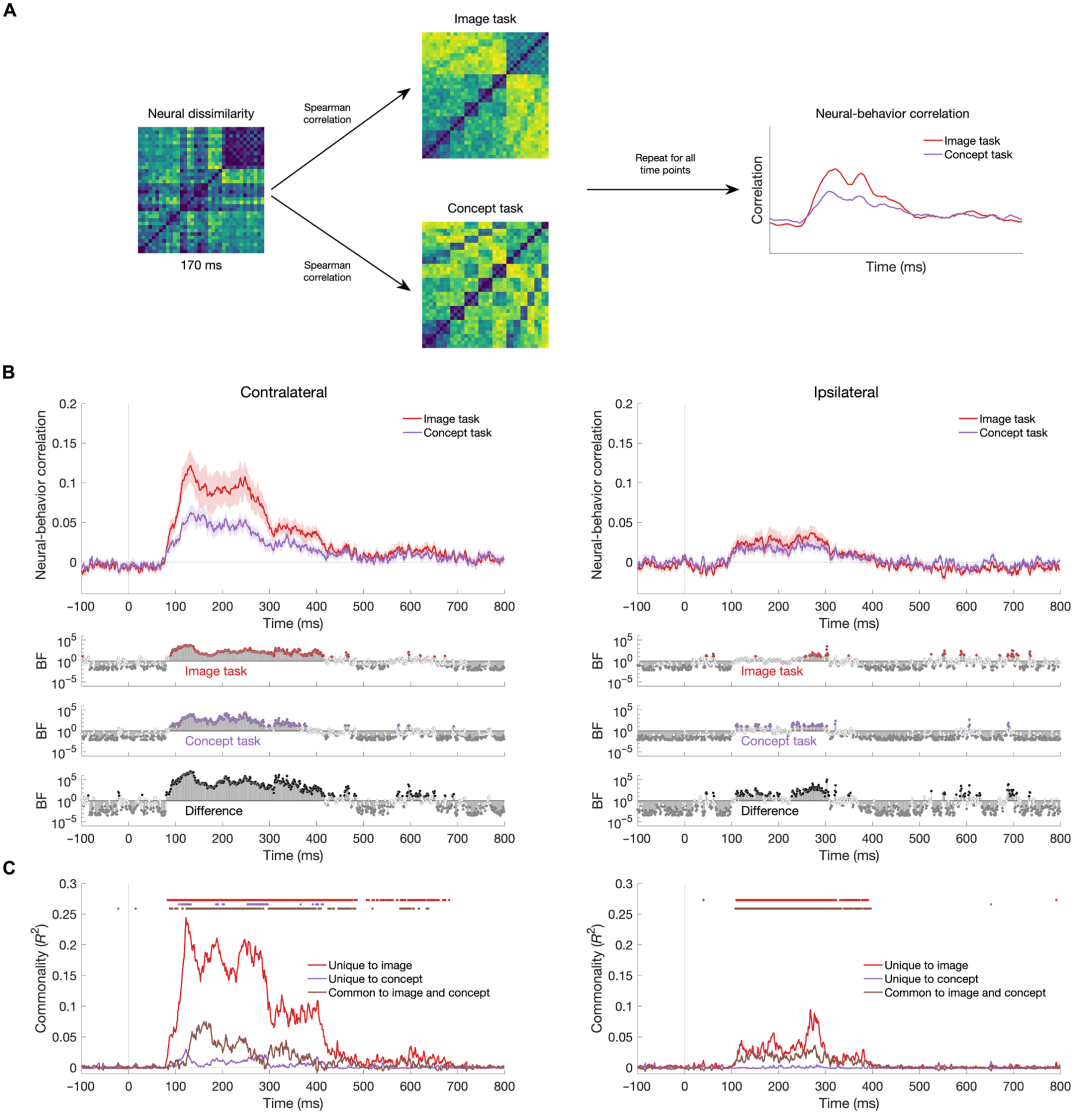
Neural-behavior relationship for image and concept tasks. (**A**) Explanation of how neural-behavior correlations were calculated. For each hemisphere and visual field condition, the neural dissimilarities across all 36 images were correlated with the behavioral dissimilarities for the image task and the concept task, separately for each neural time point following image onset. The mean was subsequently calculated for the contralateral and ipsilateral conditions per behavioral task (example results plot shown). (**B**) Neural-behavior correlations across time for contralateral (left) and ipsilateral (right) stimulus presentations, collapsed across the left and right hemispheres. Behavior from the image and concept tasks was reliably reflected in the neural signal in both hemispheres, although with less fidelity in the ipsilateral hemisphere particularly for the image task. Results are shown from the single peripheral condition. (**C**) Commonality analyses. Variance in the neural data from the contralateral and ipsilateral hemispheres that was explained by the image task and concept task models and variance common to the two models. Colored dots above plots reflect time points of significant variance explained (*P* < 0.001).

Using the behavioral models constructed from the two tasks, we assessed the behavioral relevance of the representations evoked in the contralateral and ipsilateral hemispheres. We used RSA to compare the neural representations with behavioral models constructed from the image-similarity and concept-similarity versions of the odd-one-out task (Fig. 7C). The logic of these analyses is shown in Fig. 8A; neural RDMs at each time point were correlated with the image task RDM and the concept task RDM to result in a time-varying neural-behavior correlation for each task. This correlation was performed separately for the left and right hemispheres according to whether the stimuli were contralateral or ipsilateral. Figure 8B shows the mean neural-behavior correlations for contralateral and ipsilateral conditions. We found that the behavior reflected neural representations in the contralateral hemisphere (as indexed by above-zero correlations), and the neural-behavior correlation was higher for the image task than the concept task. In contrast, the neural-behavior relationship was similar for the image and concept tasks in the ipsilateral hemisphere, with greater statistical validity for the concept task.

To complement the neural-behavior correlations, we performed a commonality analysis. This analysis, similar to that in (*31*), assessed how each behavioral model was associated with unique variance in the neural data and the variance common to both models. Specifically, we performed linear regression on the neural RDMs using the image and concept behavioral models as predictors and compared it to linear regression models with each behavioral model alone. The differences in *R*^2^ between models revealed the unique variance in the neural data that was related to the image and concept models, as well as the variance common to both models (*32*). These calculations of commonality were compared to distributions calculated from 1000 repeats using random permutations of the neural RDMs. Figure 8C shows the commonality estimates in the contralateral and ipsilateral hemispheres. Time points with commonality estimates that exceeded all permutations are marked on the plot (*P* < 0.001). In both the contralateral and ipsilateral hemispheres, there was robust unique variance associated with the image model. The concept model was minimally but reliably reflected in the contralateral, but not ipsilateral, neural signal. Variance common to both models was notable in both hemispheres. Crucially, the common variance accounted for a much larger proportion of the total variance in the ipsilateral hemisphere than the contralateral hemisphere. Together, these results indicate a fundamental difference in the types of information represented by the contralateral and ipsilateral hemispheres. Specifically, this suggests that information transfer from the contralateral to ipsilateral hemisphere might prioritize information that reflects object meaning; this likely includes certain visual features that are characteristic of object concepts but not image statistics that are less relevant to object identity.

## DISCUSSION

In this study, we investigated the temporal dynamics of peripheral object processing within the left and right hemispheres. Across two different peripheral stimulus conditions, we found that image representations traverse the hemispheres across multiple stages of processing. Stimulus information was initially biased toward the contralateral hemisphere, but subsequent stages of processing showed robust neural responses in both hemispheres. Dynamics were different in the left and right hemispheres, regardless of whether the images were contralateral or ipsilateral. Specifically, the right hemisphere seemed to derive stimulus representations with higher fidelity than the left hemisphere, independent of visual field. RSA revealed that information is shared between the contralateral and ipsilateral hemispheres with a delay around 15 to 30 ms. Last, we found evidence that representations in both hemispheres correlate with behavior on stimulus similarity tasks. While the contralateral hemisphere was more strongly correlated with image-related judgments than concept-related judgments, the ipsilateral hemisphere showed little difference in behavioral relevance for the two tasks. Together, these results suggest that hemispheric transfer prioritizes object category information, which reflects both meaningful image features and object meaning, over image statistics that are not informative to meaning.

To our knowledge, this is the first study to assess representational dynamics in the left and right hemispheres in humans. We provide strong evidence of contralateral dominance of perceptual information in the visual system. This adds to previous work documenting contralateral dominance in the strength of neural activation (*10*–*12*) by showing that the contralateral hemisphere also represents visual information with higher fidelity than the ipsilateral hemisphere. Specifically, we found stronger and earlier representations for contralateral visual field relative to ipsilateral visual field stimuli. Yet, there were still relevant representations for ipsilateral stimuli, indexed by above-chance decoding, indicating that information was transferred from the contralateral to ipsilateral hemispheres. This remained true even for the dual peripheral condition: Each hemisphere contained information about the two stimuli that were presented simultaneously, regardless of whether they were in the contralateral or ipsilateral visual field. Our task was irrelevant with respect to the visual stimuli, so the sharing of information across hemispheres appears to be a fundamental feature of neural processing rather than a goal-driven process.

We found evidence for interference of information processing for two simultaneously presented peripheral images. Specifically, there was a reduction in information in the dual relative to single peripheral condition in both the contralateral and ipsilateral hemispheres but only after initial stages of processing (later than 150 ms). Thus, the first stages of processing seem to proceed unimpeded, likely within early visual regions with strong retinotopic organization. Hemispheric transfer then seems to affect subsequent processes within mid- to high-level regions that code for objects and are less retinotopic. Previous EEG–functional magnetic resonance imaging work showed that high-level extrastriate regions respond to stimuli from both visual fields, whereas low-level extrastriate regions respond primarily to the contralateral hemifield (*33*). We found that the difference in decoding between the single and dual image conditions was more robust in the ipsilateral hemisphere, which points to these later, high level processes being more subject to interference. It should be noted that the simultaneous stimuli in the current study were presented in symmetrical positions relative to the vertical midline, and it could be that interference is particularly sensitive to this configuration. Nevertheless, the timing of the interference effects highlights some possibilities about the nature of different processing stages, namely regarding temporal multiplexing. A growing body of literature has revealed that multiple rapidly presented images are represented simultaneously in the brain (*34*–*36*), yet the temporal resolution of processing decreases from low-level to higher-level regions in the visual hierarchy (*37*, *38*). In the context of peripheral image processing, a possibility is that early retinotopic brain regions quickly and process input from the contralateral hemifield serially and then transfer information to higher regions within both hemispheres. These successive brain regions have longer temporal integration windows, which could plausibly lead to interference between different stimuli (e.g., masking) but would also enable integration when stimuli presented temporally or spatially adjacent are contextually related.

EEG is not known for its high spatial resolution, but we implemented several strategies to ensure that the analyses were assessing distinct information from each hemisphere. First, we used clusters of electrodes that were spatially distinct, over the left and right occipitotemporal regions. Electrodes in these regions have previously been used to document hemispheric differences for face and word perception (*39*–*41*). Second, we performed a Laplacian transformation on the neural responses to highlight local patterns of activity, enhancing the spatial resolution of the signals (*42*). The results indicate that there were distinct sources per hemisphere. The sensor searchlight analyses depict clear contralateral sources of information, with weaker ipsilateral clusters at later time points (see the Supplementary Materials). Last, our time resolved decoding results show that the dynamics look distinct in the two hemispheres (e.g., with a later peak for ipsilateral stimuli). If there was only one neural source of information, then we would expect an overall reduction for ipsilateral responses without a change in the timing.

In addition to varying dynamics for contralateral versus ipsilateral representations, the results of this study highlight differences between the left and right hemispheres of the brain. In particular, the left and right hemispheres seem to retain hemispheric-specific dynamics regardless of whether they are contralateral or ipsilateral, which is evidence that each hemisphere is specialized for certain types of information, and processing is distributed for these different processes. Furthermore, there was a consistent trend in which the right hemisphere had stronger representations than the left. Given the types of stimuli used in the current study, one could surmise that the hemispheric differences we observe are driven by the categories of words, which tend to be lateralized to the left hemisphere (*43*), and faces, which are lateralized to the right hemisphere (*44*). However, we found that even when excluding faces and words from the analysis (objects only), there was still a right hemispheric dominance in the strength of decoding. For faces and words separately, decoding was also numerically stronger in the right hemisphere than the left hemisphere, but this was not statistically reliable, possibly due to fewer images in each category leading to lower power (see the Supplementary Materials). Together, this hints toward more general hemispheric processing differences, potentially centered around biases for specific visual features. It has been postulated that the two hemispheres contain distinct subsystems for abstract and specific visual object recognition (*45*, *46*), with the right hemisphere more effective for specific exemplar recognition, so one possibility is that the focus on stimulus information in the current study inadvertently biased toward a right hemispheric advantage. However, it should be noted that the hemispheric neural-behavioral analyses showed stronger correlations for the image task in the right hemisphere, but there was no left advantage for the concept task, a task that likely required a more abstract recognition strategy.

Despite hemispheric differences, there was evidence of clear representational overlap between the hemispheres. Our results show that the ipsilateral hemisphere represents stimulus information similarly but after a delay relative to the contralateral stimulus. Our estimates of the delay were on the order of 15 to 30 ms. This again points to clear evidence of information transfer from one hemisphere to the other. More support for this transfer delay is evident from the delay of decoding onset for ipsilateral relative to contralateral stimuli (see Table 1). Beyond the timing differences, the similarity in the structure of stimulus representations between the hemispheres contralateral and ipsilateral to the experimental stimuli suggests an element of redundancy in the system. The ipsilateral hemisphere receives peripheral visual information indirectly, via the contralateral hemisphere, so shared or redundant information does not come as a surprise. Yet, this indirect transfer does not preclude different processes being carried out on inputs to the contralateral and ipsilateral hemispheres. We found that the information within the hemisphere ipsilateral to the stimulus did not contain any unique information relative to the contralateral hemisphere, but rather the information was largely duplicated throughout the time course of processing. However, the specific information that was transferred to the ipsilateral hemisphere was only a subset of that represented within the contralateral hemisphere. This raises the possibility that hemispheric transfer operates as a filtering mechanism, modulating neural activity to focus the most important types of information. Evidence suggests that cross-hemispheric neurons are mainly excitatory, but they target both excitatory and inhibitory neurons (*47*), which seems a plausible route for information pruning. So, despite the apparent redundancy across the hemispheres, perhaps the ipsilateral hemisphere plays an important role in being able to process only the most relevant information.

Our analyses into the behavioral relevance of the neural representations support this “filtering via hemispheric transfer” theory. The contralateral hemisphere was biased toward perceptual over conceptual judgments, with behavioral responses on the image task correlating more strongly with the neural signal than responses on the concept task. Yet, in the ipsilateral hemisphere, there was little difference between the behavioral relevance for perceptual and conceptual judgments. Behavioral responses in the image task and the concept task, while separable, were highly correlated, so we would not expect them to necessarily dissociate. The commonality analysis revealed reliable variance common to the two behavioral models in both the contralateral and ipsilateral hemispheres. We suggest that this common variance reflects judgments that are similar between the two tasks, which seem to be object-category focused. In contrast, the types of information that were lost to hemispheric transfer mainly related to perceptual judgments, likely related to image statistics. These findings indicate that hemispheric transfer might prioritize information relating to object identity; this includes object meaning as well as image features that are characteristic of object identity (e.g., trees tend to be green).

There are still many open questions about the nature of hemispheric functioning and hemispheric transfer. Here, we only tested stimuli with a fixed eccentricity along the horizontal meridian, but hemispheric processing might vary with the location and eccentricity of stimuli in the visual field; for example, increasing eccentricity might increase the hemispheric delay. Testing stimuli in various locations across the visual field might give clues into the timing and content of perceptual processing within each hemisphere. It would also be interesting to probe the degree to which semantic information is extracted from peripheral stimuli in such paradigms and its representation in each hemisphere. Relatedly, investigating semantics might help disentangle the specific types of information that are prioritized for transfer across the hemispheres, for example, using tasks that dissociate information about image features from those of conceptual content. The current study did not focus on semantic integration, but the current paradigm could also be used to assess how the brain extracts perceptual and semantic information in naturalistic scenes, particularly in the case of congruent or incongruent information across the hemifields. Overall, we think that this is a promising line of research with exciting possibilities in investigating how the left and right hemispheres of the brain work together for perception.

## MATERIALS AND METHODS

### Participants

Participants were 20 adults recruited from the University of Sydney (15 females; median age, 22 years) in return for payment or course credit. This sample size is similar to previous work on contralateral visual responses (*18*) and the temporal dynamics of visual processing (*34*, *36*). The study was approved by the University of Sydney ethics committee (approval #2016/849), and informed consent was obtained from all participants. Edinburgh handedness scores indicated that 17 participants were right-handed, 1 was left-handed, and 2 were ambidextrous. The same trend in results was seen if only right-handed individuals were analyzed (see the Supplementary Materials). All participants reported normal or corrected-to-normal vision.

### Stimuli and design

Participants viewed images that appeared centrally or to the left or right of fixation on a 1920 × 1080 resolution ASUS VG236 monitor, while their neural activity was measured with EEG. There were 36 stimuli: four different image exemplars of six basic objects: fish, bird, face, boat, tree, and tool; and word labels for the same six objects in lower- and uppercase letters (Fig. 1A). Half the trials were words, and half were objects. Stimuli were presented at approximately 3.24° × 3.24° of visual angle and at an eccentricity of 3.24° to the center of the object. All stimuli were presented using PsychoPy (*48*). We intended to present the words in italics as well as original typeface, but an unforeseen issue with the PsychoPy presentation meant that the italics were not applied, so each specific word stimulus was presented double the number of times compared with each object (but only half of these were analyzed; see below).

There were three types of experimental sequences: central, single peripheral, and dual peripheral (Fig. 1B). These sequences were interleaved throughout the experimental session. Here, we focus only on the peripheral and dual peripheral sequences as these inputs are projected to a single hemisphere in a bottom-up fashion. In each sequence, there were 192 trials. Each trial was presented for 100 ms with a gap of 100 ms between trials (5-Hz presentation). In the single peripheral condition, stimuli were presented half to the left and half to the right of fixation equiprobably and in random order, with only one stimulus presented at a time. Each sequence consisted of four repeats per object stimulus (two on the left and two on the right) and eight repeats per word stimulus (four left; four right). In the dual peripheral condition, every trial consisted of one stimulus to the LVF and one to the RVF simultaneously. There were 96 images and 96 words on each side in random combinations. Within each sequence, trials were presented in random order.

Across the experiment, there were 24 single peripheral and 12 dual peripheral sequences, which, in total, contained the same number of stimulus repeats per visual field. In sum, there were 48 repeats of each of the 24 image stimuli and 96 repeats of each of the 12 word stimuli in each hemifield for each condition. To maintain equal trial numbers per class, only 48 repeats for the word stimuli were analyzed, randomly selected.

During the experimental session, participants were asked to monitor the three dots (central fixation, left, and right) marking the possible stimulus locations and detect when one turned red (Fig. 1B) and indicate detection by button press. Participants were asked to maintain fixation on the central dot. This task was designed to be orthogonal and irrelevant to the stimuli. The fast stimulus presentation, random image sequences, and the orthogonal task reduced the likelihood of participants moving their eyes in a stimulus-specific manner. Yet, the task ensured that participants maintained attention across the whole visual field throughout the experiment, regardless of stimulus presentation condition.

### EEG recording and preprocessing

EEG data were continuously recorded from 64 electrodes arranged in the international 10-20 system for electrode placement (*49*) using a BrainProducts ActiChamp system, digitized at a 1000-Hz sample rate. Scalp electrodes were referenced to Cz during recording. The EEGLAB toolbox (*50*) was used to preprocess the data offline. First, we interpolated electrodes that exceed 5 SDs of kurtosis, and then a common average reference was applied. We filtered the data using a Hamming windowed sinc finite impulse response (FIR) filter with highpass of 0.1 Hz and lowpass of 100 Hz as in our previous work (*34*, *36*). Downsampling was not applied, so the temporal resolution of the data was 1 ms. Epochs were created for each stimulus presentation ranging from −100 to 800 ms relative to stimulus onset. Last, we used the current source density (CSD) toolbox (*51*) to perform a Laplacian transformation (*42*) which calculates the second spatial derivative of the scalp potentials. This transformation enhances the spatial resolution of the EEG signal at the scalp, in our case ensuring greater distinction between left and right hemispheric responses.

### Neural decoding

To investigate the neural representations of lateralized stimuli in the two hemispheres, we used MVPA to assess stimulus-specific representations per hemifield (left/right), hemisphere (left/right), and presentation condition (single/dual peripheral). Given that EEG responses are typically stronger for contralateral stimuli (*12*), we expected that stimuli would also be represented with higher fidelity in the contralateral hemisphere than the ipsilateral hemisphere and potentially represented with different dynamics. Further, we were interested in how conflicting information to the hemispheres (as in the dual peripheral condition) influenced the neural representations relative to the single peripheral condition.

EEG data were analyzed using time-resolved classification methods and implemented using the CoSMoMVPA toolbox (*52*). Decoding was performed separately for the left and right hemispheres using clusters of six occipito-temporal electrodes (Fig. 1C). The electrodes chosen were O1, PO3, PO7, P3, P5, and P7 for the left cluster and O2, PO4, PO8, P4, P6, and P8 for the right cluster. These clusters encompass similar electrodes to previous work investigating lateralized responses (*39*–*41*) but containing sufficient electrodes to allow for pattern analysis. The trend in decoding across conditions did not appear dependent on the specific electrodes chosen; see the Supplementary Materials for decoding with larger clusters encompassing more anterior electrodes. Decoding was performed separately for each participant, for each presentation condition (single/dual × left/right hemifield), and for each single time point in the epoch (1-ms time resolution). Data were pooled across the six EEG sensors in each cluster, and we tested the ability of a linear discriminant analysis classifier to discriminate between the patterns of neural responses associated with each stimulus. A 12-fold cross-validation procedure was used, with each fold containing independent trial sequences. All pairs of combinations for the 36 stimuli (e.g., face1 versus tree2 and word-tool1 versus fish4) were decoded, resulting in 630 unique contrasts across time per condition per participant. Classifier accuracy was calculated as the proportion of correct classifier predictions across all folds, and the group mean was calculated per condition. All decoding contrasts were pairwise, so chance performance was 0.5. Accuracy of classifier predictions reflected the information in the neural signal, where above-chance classification accuracy (>0.5) indicated that the hemisphere contained information about the stimuli.

### Behavioral similarity task

We were also interested in how the neural responses within each hemisphere related to behavioral judgments for the same stimuli. In two online experiments (*53*), conducted independently of the EEG acquisition, new groups of participants rated the similarity between the experimental stimuli using a triplet odd-one-out task (*54*, *55*) with the 36 experimental stimuli.

Participants were undergraduate students from the University of Sydney who participated in return for course credit. The experiments were programmed in jsPsych (*56*) and hosted on Pavlovia (*48*). In each experiment, there was a separate set of instructions: choose the one that looks different (*N* = 21) or choose the one that is conceptually different (*N* = 21). On each trial, three experimental stimuli were presented simultaneously, and participants were asked to choose the odd one out by clicking on the stimulus (Fig. 2A). There were 400 trials in the experiment, and stimulus combinations were randomly chosen.

Behavioral responses were used to construct RDMs for each task. For each trial, we calculated dissimilarity across the pairs of stimuli (three pairs for the three distinct stimuli). The chosen odd-one-out stimulus was coded as dissimilar from each of the other two stimuli (value of 1), and the two other stimuli were coded as similar (value of 0). Across all trials of all participants, the dissimilarity of each stimulus pair (e.g., face1 versus word-tree2) was calculated as the mean response for all trials in which those two stimuli were presented together, a measure of their relative similarity to each other compared with the other stimuli in the set.

### Representational similarity analyses

To investigate the relationship in the structure of stimulus representations between the two hemispheres, we used RSA (*30*). RSA allowed a comparison between hemispheres which was abstracted away from specific neural activity patterns and rather focused on the relationships between stimulus representations. In this case, RSA allowed hemispheric-specific representations to be compared with representations of the other hemisphere in the single and dual peripheral conditions. In a subsequent set of analyses, we used RSA to compare representations within each hemisphere with behavioral judgments, to assess the content of information within each hemisphere.

Using the neural and behavioral results, we constructed RDMs, which quantified the similarity between each stimulus (e.g., Fig. 5A). Each of these RDM models was a 36 × 36 matrix of dissimilarity for each of the 36 stimuli with each other stimulus, using the relevant neural or behavioral measure. The RDMs were symmetrical across the diagonal, with 630 unique values. Neural RDMs used decoding accuracy for each pair of stimuli at each time point (1-ms temporal resolution). Separate 36 × 36 stimulus RDMs were constructed for each hemisphere, time point, and participant, where each cell contained the mean decoding accuracy between two stimuli. The behavioral RDMs were based on the group mean dissimilarity scores from the two behavioral experiments. We also constructed two additional stimulus models based on the stimulus category (image/word) and concept (e.g., tree, tool, and face).

Using RSA, we investigated how representations varied across the hemispheres. First, we correlated the left and right hemisphere RDMs using Spearman correlation to assess similarity of the lower diagonals of the RDMs (i.e., the unique pairwise values), for every pair of time points. This allowed us to assess how representations were similar across the hemispheres, and whether this similarity was dependent on transfer delays. Last, we correlated the neural models across time with each behavioral model to assess how neural information might inform overall perception. Correlations were performed for each EEG participant separately, and the mean was calculated across the group.

For any analyses comparing hemispheres, we used a split-half comparison method to reduce spurious correlations due to correlated noise. Specifically, we constructed two RDMs per condition, based on odd or even sequences (i.e., using sixfold cross-validation to decode stimulus pairs). We then assessed similarity in neural RDMs across hemispheres by using different sequences; for example, comparing the left hemisphere RDM from odd sequences with the right hemisphere RDM from even sequences and vice versa and then taking the mean.

### Statistical testing

To assess neural representations within the hemispheres, we used Bayesian statistics to determine the evidence for the alternative relative to the null hypotheses (*57*–*61*). For decoding analyses, the alternative hypothesis of above-chance (50%) decoding was tested. For correlation analyses, the alternative hypotheses of above- and below-zero correlations were tested. We used the “BayesFactor” package in R (*62*). BFs were calculated using a Jeffreys-Zellner-Siow (JZS) prior, centered around chance decoding of 50% (*60*) with a default scale factor of 0.707, meaning that for the alternative hypotheses of above- and below- chance decoding, we expected to see 50% of parameter values falling within −0.707 and 0.707 SDs from chance (*59*, *60*, *63*, *64*). A null interval was specified as a range of effect sizes between −0.5 and 0.5 (*65*).

A BF is the probability of the data under the alternative hypothesis relative to the null hypothesis. We consider BF > 3 as evidence for the alternative hypothesis (above-chance decoding and reliable correlations). To calculate the onset of effects, we used a conservative estimate of the first time that there was sustained evidence for 10 ms (10 consecutive time points with BF > 10). We interpreted BF < 1/3 as evidence in favor of the null hypothesis (*59*, *66*).
