# Supplementary material for: Dynamics of visual object coding within and across the hemispheres: Objects in the periphery
Source: Sci Adv. 2025 Jan 1;11(1):eadq0889. doi: 10.1126/sciadv.adq0889 (PMC11691691; doi:10.1126/sciadv.adq0889)
Supplement: Supplementary file 1 — Supplementary Text Figs. S1 to S14 Table S1 [file sciadv.adq0889_sm.pdf]

Science Advances
AAAS

Supplementary Materials for

Dynamics of visual object coding within and across the hemispheres: Objects in the periphery

Amanda K. Robinson *et al.*

Corresponding author: Amanda K. Robinson, amanda.robinson@uqconnect.edu.au

Sci. Adv. **11**, eadq0889 (2025)
DOI: 10.1126/sciadv.adq0889

This PDF file includes:

Supplementary Text
Figs. S1 to S14
Table S1

Supplementary Text

Target detection performance

During the task, participants detected colour change targets that appeared throughout the sequence. These targets could appear to the left, right or centre of the monitor, in the positions of the possible stimulus locations. The proportion of hits according to target position and experimental condition is shown in Figure S1.

Information topographies

Searchlight decoding was conducted to examine which clusters of electrodes had the highest decoding accuracy. Figure S2 shows that left hemifield stimuli had highest decoding over the right hemisphere and right hemifield stimuli had highest decoding over the left hemisphere. This validates our a priori cluster selection, showing that most information was contained in the left and right electrode clusters we chose.

Decoding using different clusters

To show that the results still hold with larger electrode clusters encompassing more anterior electrodes, we ran the decoding analyses again using clusters double the size. The electrodes included were the six originals (O1, PO3, PO7, P3, P5, P7 for left hemisphere, or right hemisphere equivalents) as well as six more anterior electrodes (CP3, CP5, TP7, TP9, C3, C5 and T7 for left hemisphere, or right hemisphere equivalents). Figure S3 shows the mean decoding accuracy by electrode cluster and stimulus position for the single-peripheral condition, and Figure S4 is decoding in the dual-peripheral condition. As can be seen, the results are very similar to those from the smaller clusters shown in the manuscript, thus validating our original choice of electrodes.

We also conducted frontal decoding in an equivalent way to the posterior decoding – using frontal channels from the left and right sides separately: AF7/AF8, F5/F6, F7/F8, FC5/FC6, FT7/FT8, FT9/FT10. The results (see Figure S5) show minimal above chance stimulus decoding using these two clusters. Furthermore, the time periods that show some evidence for decoding, particularly for the right frontal cluster, are later than the times in the posterior decoding (as shown in Figures 2 and 3 of the manuscript), likely reflecting stimulus-specific activity in frontal cortex rather than stimulus-specific eye movements to peripheral stimuli.

Image decoding according by object class

To assess how decoding accuracy varied across the different object classes in our stimulus set, we calculated classification accuracy for peripheral stimuli separately for objects, faces and words. Mean pairwise decoding is shown for the 20 non-face objects (Figure S6), 4 face stimuli (Figure S7), and 12 word stimuli (Figure S8). Each plot shows broadly the same dynamics as for the whole stimulus set (shown in the manuscript Figure 3), with contralateral dominance in each hemisphere. Notably, the right hemisphere superiority observed for the whole stimulus set does not appear to be driven by faces; decoding accuracy is numerically higher over the right hemisphere for non-face

objects too, indicating that the hemispheres have different computations even once accounting for stimulus class differences.

Comparison of single and dual-peripheral presentation per hemisphere

Figure S9 shows the single versus dual condition data (as in Figure 4) separated by hemisphere.

Interhemispheric representational similarity analyses

To assess how the structure of object representations varied across the hemispheres, we repeated the interhemispheric RSA restricted to the 24 object images (i.e., no words or faces). The same pattern of results was observed as the original analysis (Figure S10). We also show the original 36-stimuli analysis as in Figure 5 separated by visual field (Figure S11).

Consistency of information within and across hemispheres.

Here we show the consistency analysis in Figure 6 separated by visual field (Figure S12). The within contralateral correlations (blue lines) induced reliably larger correlations than the within ipsilateral hemisphere correlations (orange lines) and across LH-RH comparison (green lines), indicating that the contralateral hemisphere contained unique information over the ipsilateral hemisphere, but the ipsilateral hemisphere did not contain any unique information. This pattern was the same for both visual field conditions, but the within-RH comparison seemed to evoke larger correlations in both conditions.

Behavioural relevance of neural representations

The correlations between the behavioural tasks and stimulus models are listed in Table S1. We showed neural-behaviour correlations in Figure 8 that were collapsed across the left and right hemispheres; in Figure S13 we plot the correlations per hemisphere to show that the same trends apply for the left and right hemispheres. Specifically, for contralateral stimuli, the image task reflected much larger variance in the neural signal than the concept task in both hemispheres for contralateral stimuli, but this difference was reduced for ipsilateral stimuli.

Right-handers only

Three participants in our sample were either left-handed or ambidextrous. Non-right-handers are known to vary on their hemispheric lateralization relative to right handers. Decoding results from the right handers only (N = 17) reveal that the same trends apply as to the whole group (Figure S14).

Figures

Figure S1. Behavioural accuracy in detecting colour change targets, as a function of target position.

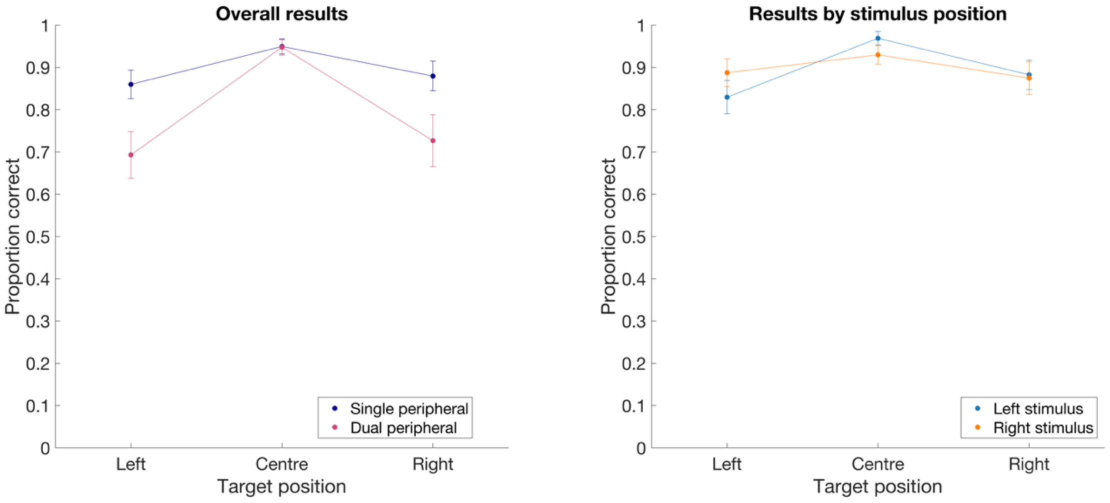

Note. Left plot shows results in detecting the colour change target according to target position and condition (single-peripheral or dual-peripheral). Right plot shows results for the single-peripheral condition according to stimulus position and target position.

Figure S2. Head maps showing decoding accuracy at different clusters of electrodes on the scalp.

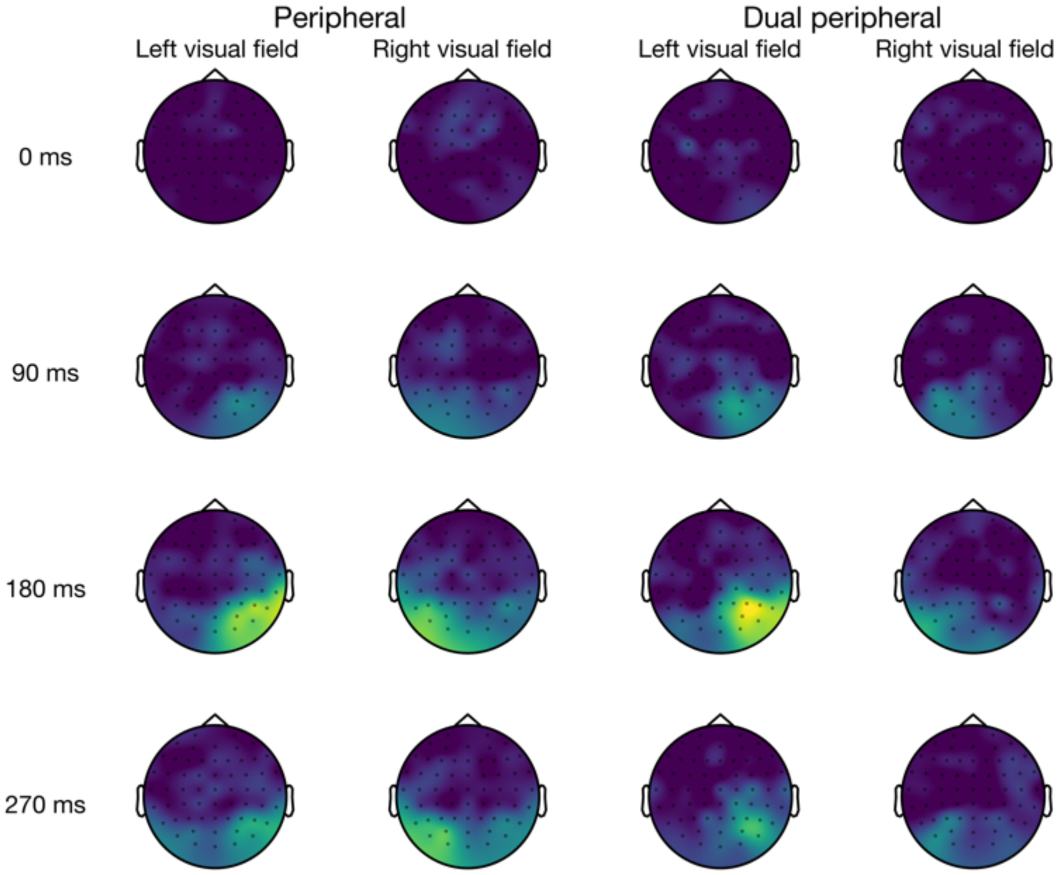

Note. Head maps show decoding accuracy for the peripheral and dual peripheral conditions, split by whether the stimuli were in the left or right visual fields, at 0ms, 90ms, 180ms and 270ms after image presentation.

Figure S3. Single-peripheral stimulus decoding using larger electrode clusters.

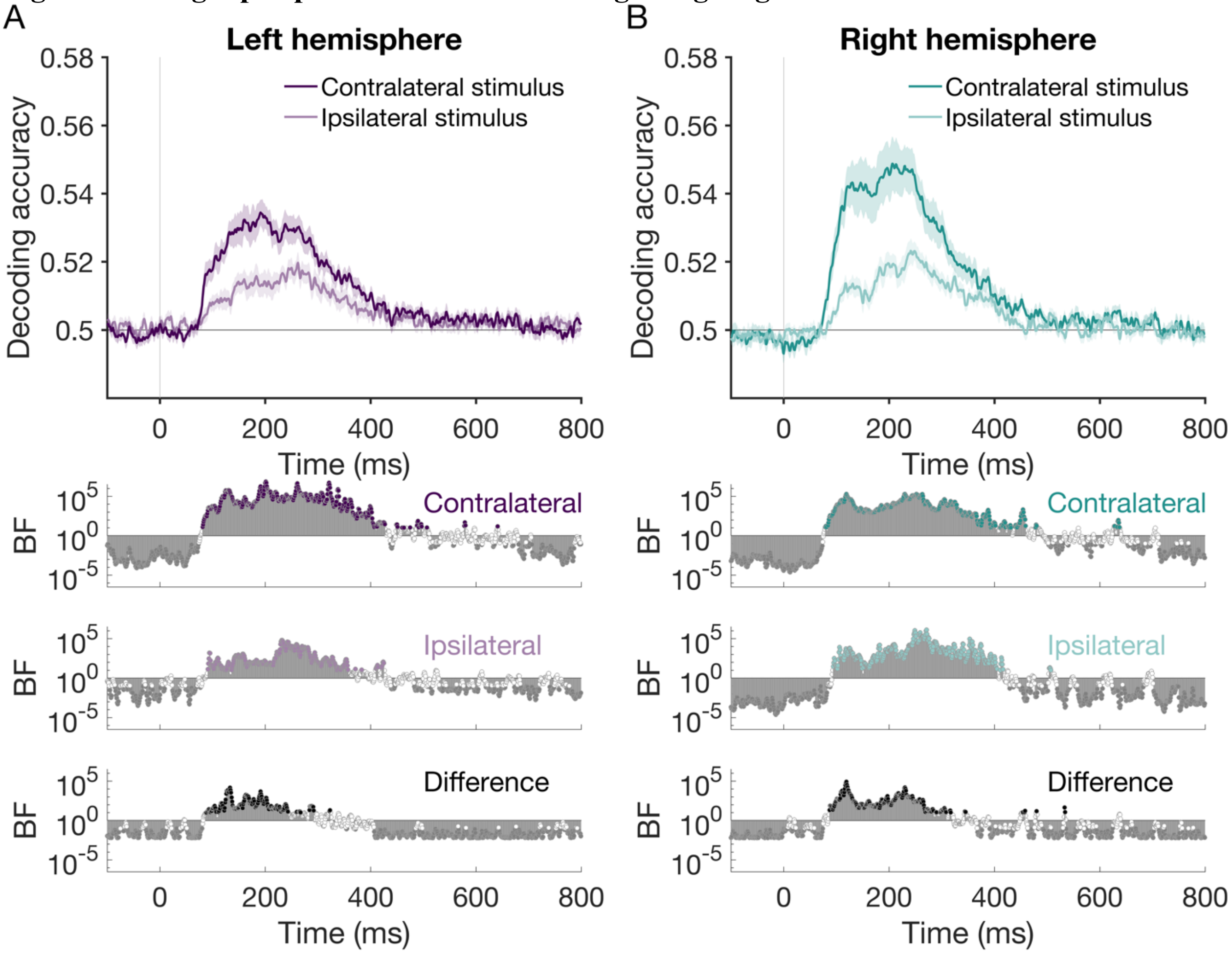

Note. Decoding was performed using 12 electrodes per electrode cluster (hemisphere). These were O1, PO3, PO7, P3, P5, P7, CP3, CP5, TP7, TP9, C3, C5 and T7 for the left hemisphere, and right hemisphere equivalents.

Figure S4. Dual-peripheral stimulus decoding using larger electrode clusters.

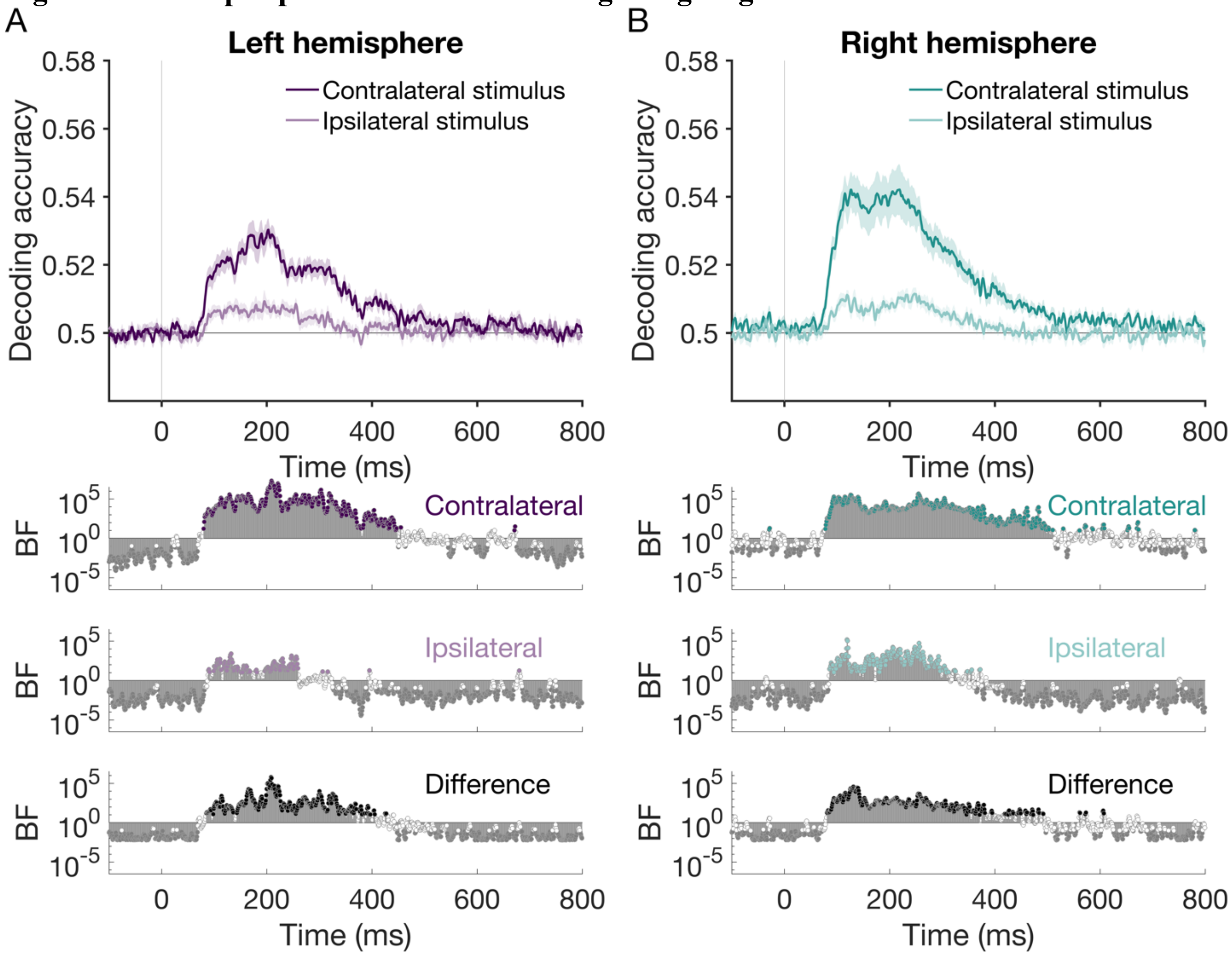

Note. Decoding was performed using 12 electrodes per electrode cluster (hemisphere). These were O1, PO3, PO7, P3, P5, P7, CP3, CP5, TP7, TP9, C3, C5 and T7 for the left hemisphere, and right hemisphere equivalents.

Figure S5. Decoding from frontal electrodes.

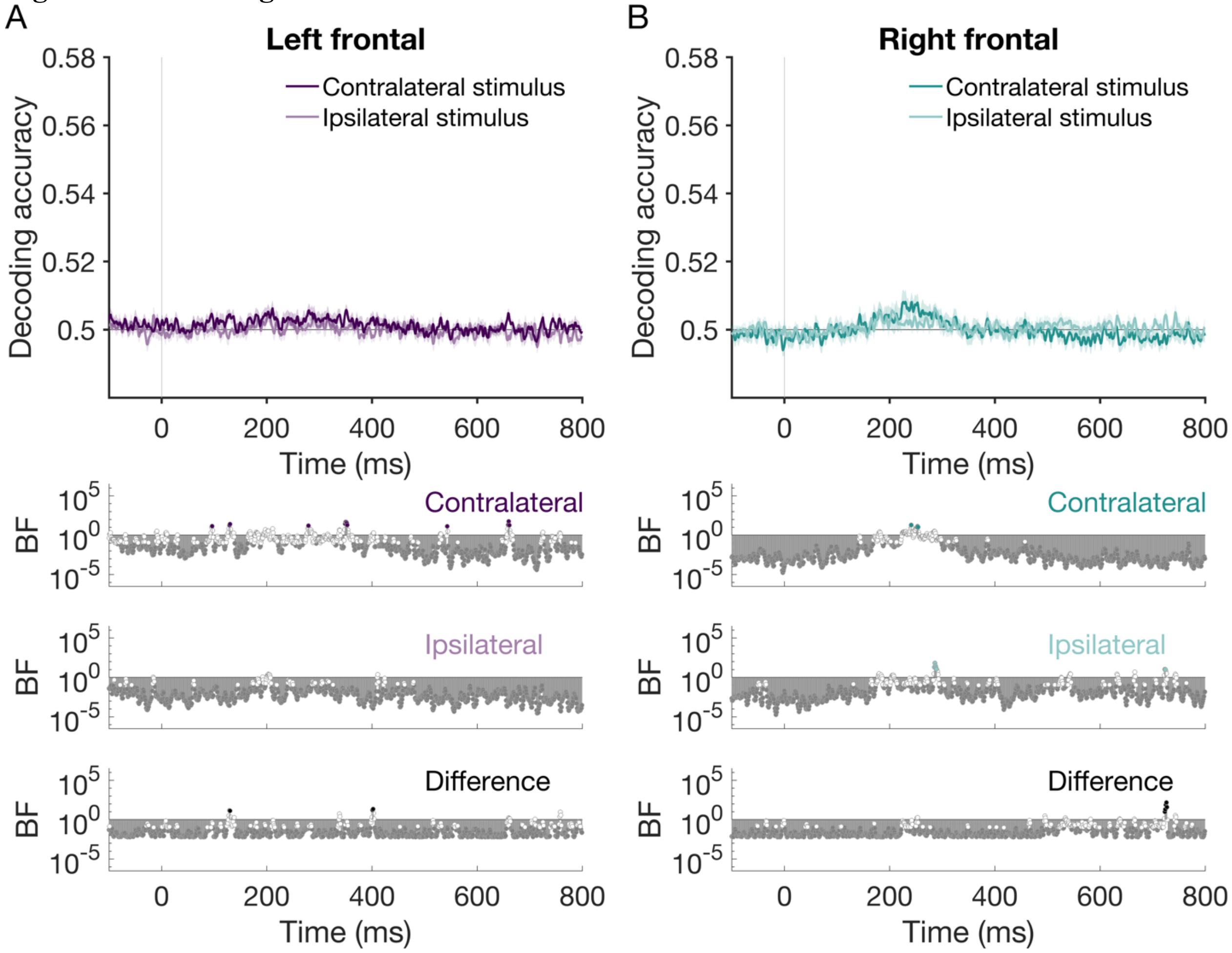

Note. The electrodes used were AF7, F5, F7, FC5, FT7 and FT9 for the left hemisphere, and their right hemisphere equivalents.

Figure S6. Decoding peripheral images for objects only (not faces or words).

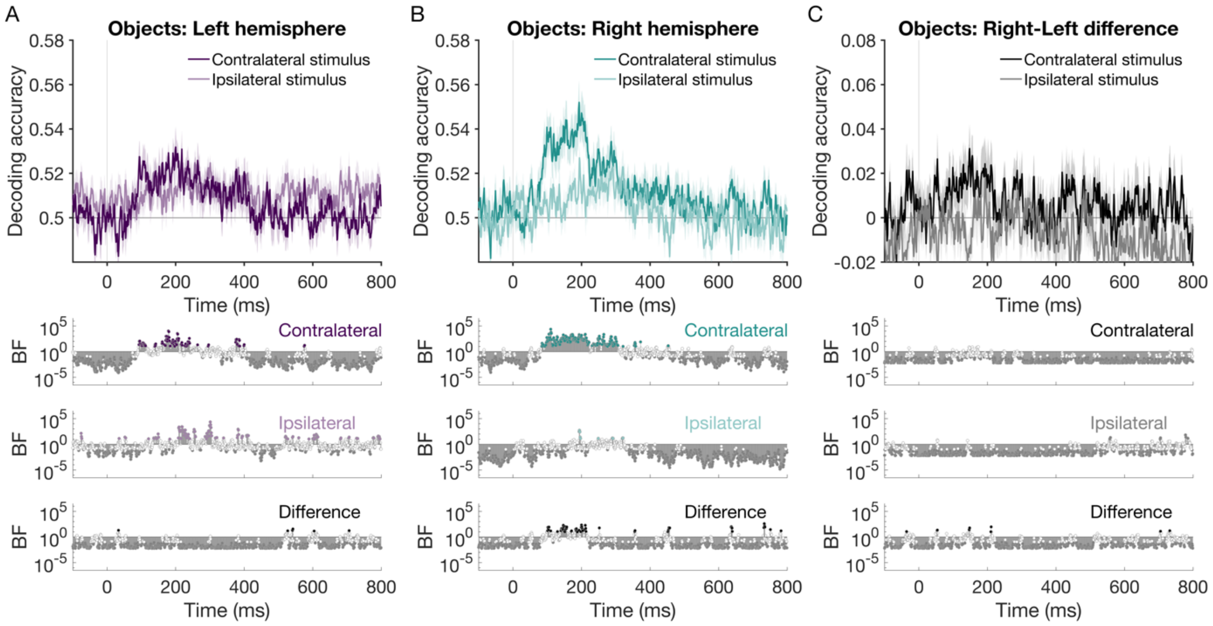

Note. Mean pairwise decoding accuracy for 20 object stimuli according to hemisphere and stimulus visual field.

Figure S7. Decoding peripheral images for faces only (not objects or words).

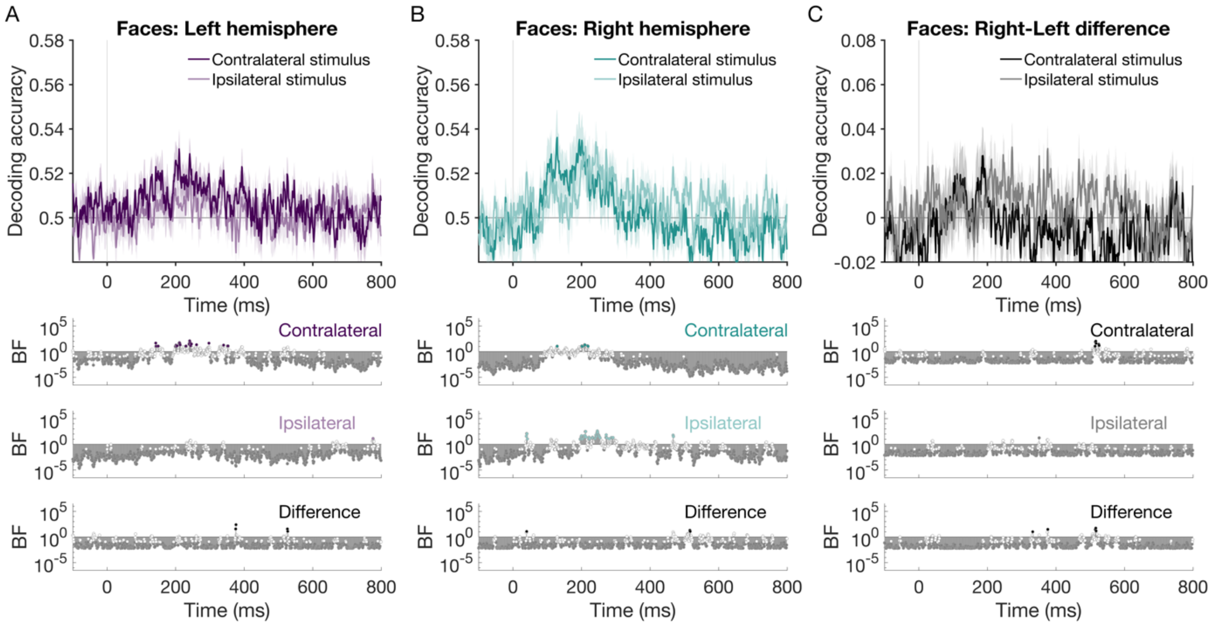

Note. Mean pairwise decoding accuracy for 4 face stimuli according to hemisphere and stimulus visual field.

Figure S8. Decoding peripheral images for words only (not faces or objects).

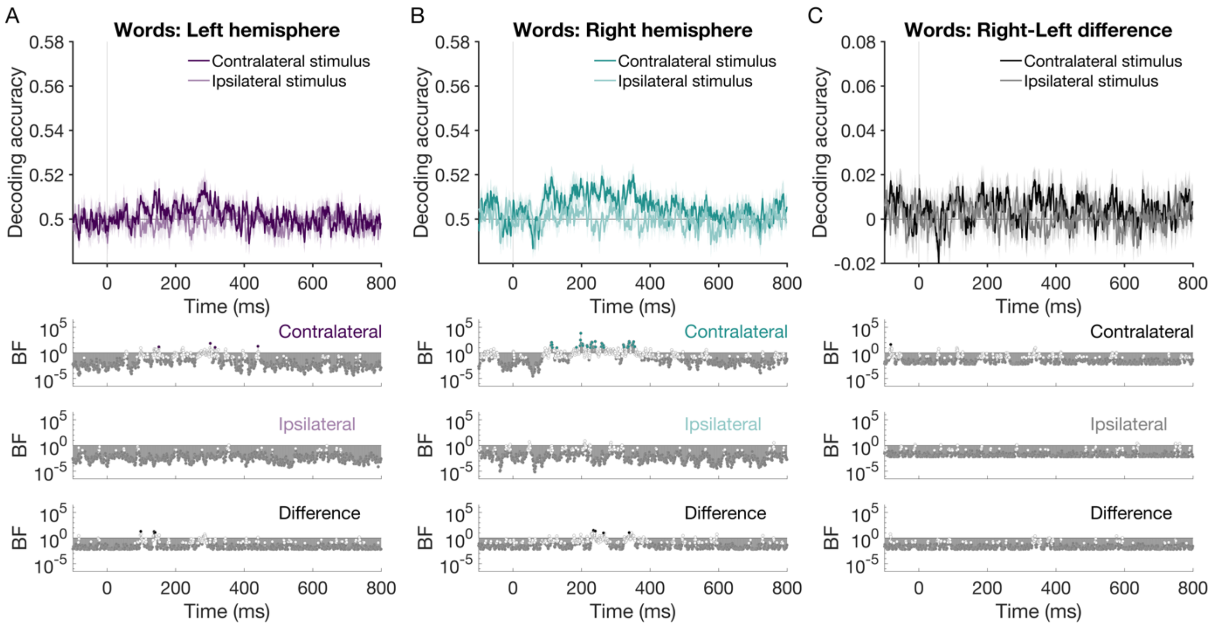

Note. Mean pairwise decoding accuracy for 12 face stimuli according to hemisphere and stimulus visual field.

Figure S9. Decoding accuracy for peripheral stimuli when presented alone (single) or with another stimulus (dual), separately for each hemisphere.

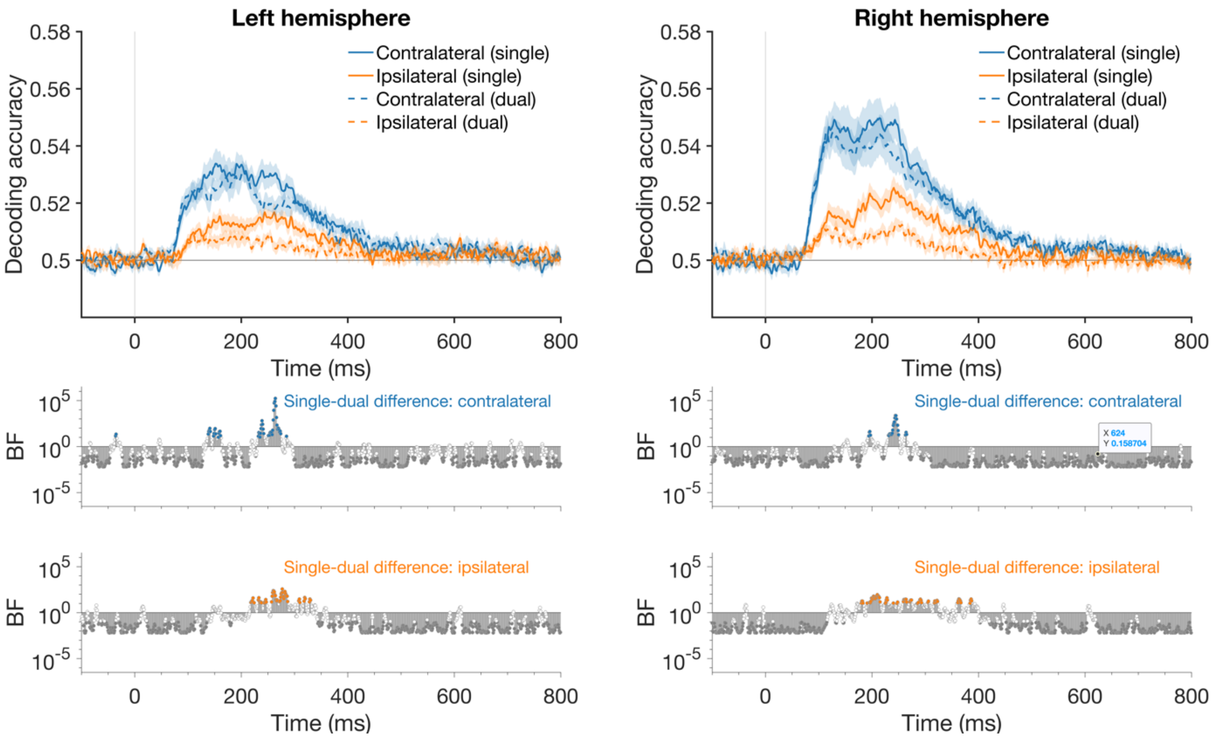

Note. Breakdown of results from Figure 4. Decoding accuracy is plotted according to whether stimuli were contralateral or ipsilateral relative to each electrode cluster. Information was stronger in the single-peripheral condition than the dual-peripheral condition (see BFs for difference), for both contralateral and ipsilateral stimuli and in both hemispheres. Shaded lines represent standard error of the mean. Bottom plots show Bayes Factors indicating the evidence for non-zero differences between single and dual conditions.

Figure S10. Shared structure of representations across the hemispheres: objects only.

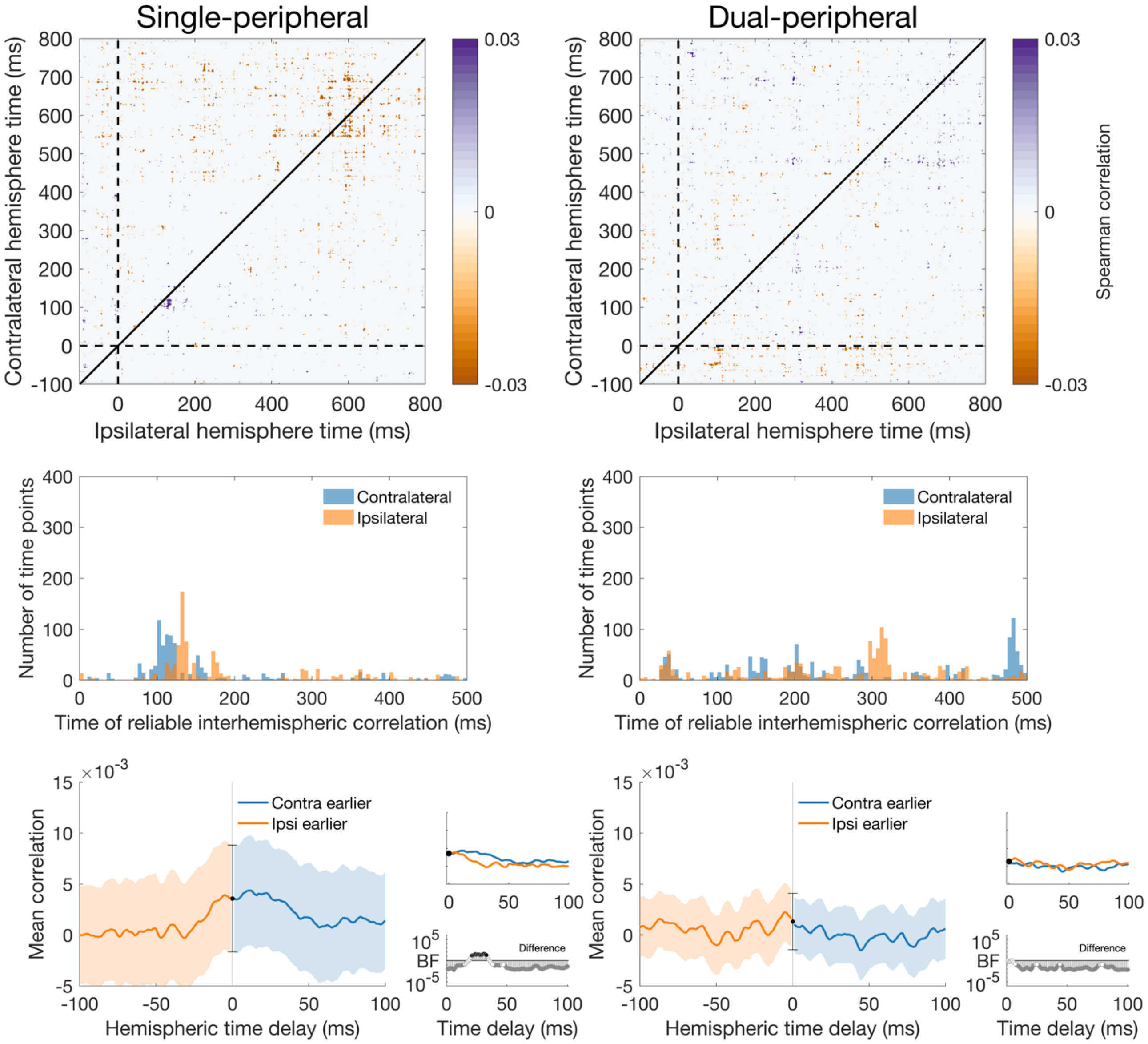

Note. Top: Plots show mean time × time correlations for representational structure in the contralateral and ipsilateral hemispheres for the single-peripheral (left) and dual-peripheral (right) conditions using 24 object stimuli only. Plots are thresholded by points with evidence for cross-hemispheric correlations different from zero (BF>3). The highest correlations were observed for contralateral to ipsilateral delays in the peripheral conditions (i.e., below diagonal correlations). Middle: Time of reliable interhemispheric correlations. Histograms show number of reliable (BF>3) positive hemispheric correlation time points as a function of contralateral and ipsilateral time. Bottom*:* Off-diagonals had higher correlations for positive ipsilateral-contralateral delays (i.e., contralateral earlier than ipsilateral) than negative delays, but only in the single-peripheral condition, indicating evidence for earlier information processing in the contralateral than ipsilateral hemisphere.

Figure S11. Shared structure of representations across the hemispheres, separately for each stimulus visual field condition.

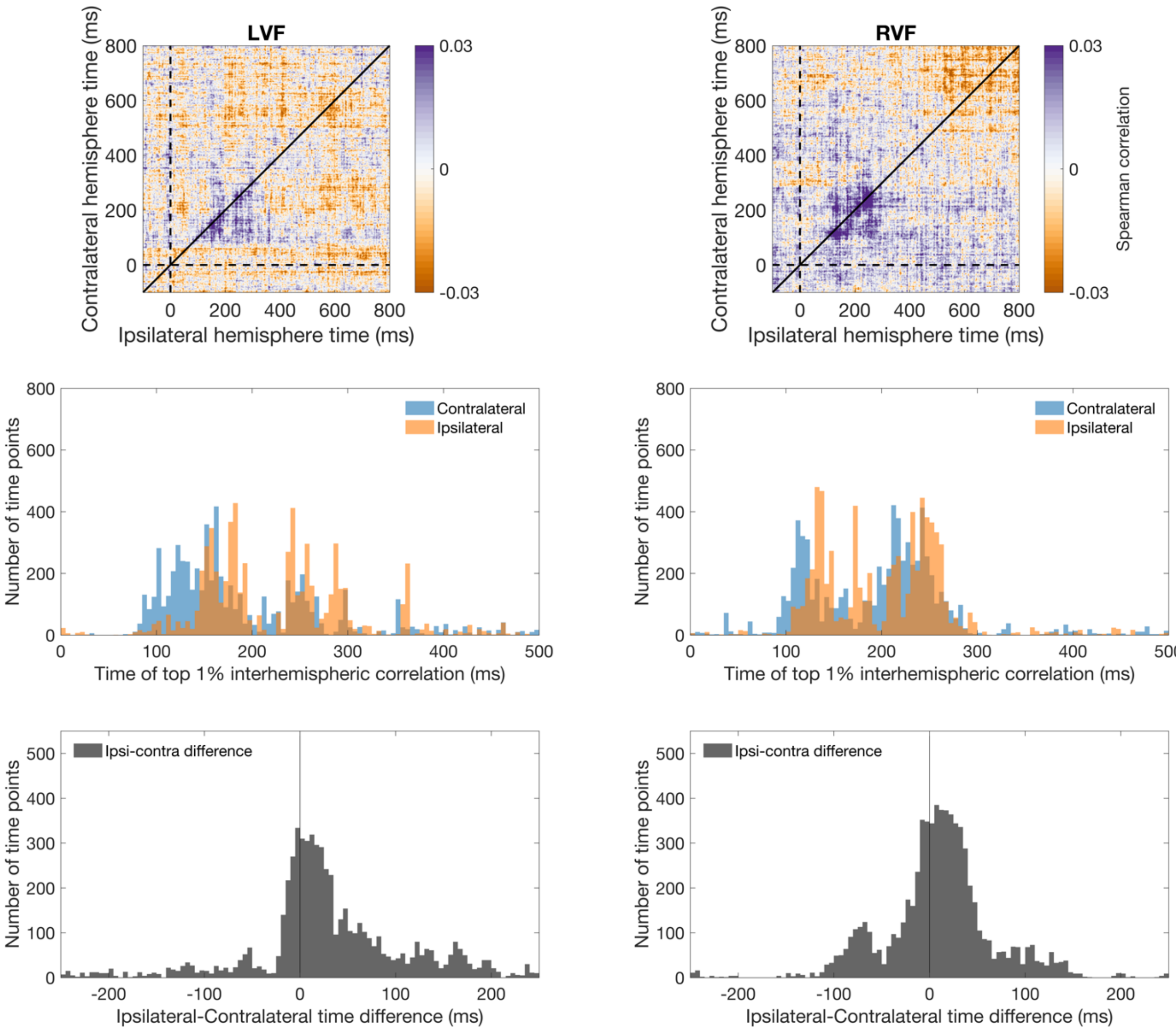

Note. A) Plots show mean time × time correlations for representational structure in the contralateral and ipsilateral hemispheres when the stimulus was in the left visual field (left) and right visual field (right) conditions. The highest correlations were observed for contralateral to ipsilateral delays in the peripheral conditions (i.e., below diagonal correlations). B) Time of top 1% interhemispheric correlations between 0 and 500ms. Histograms show number of top positive hemispheric correlation time points as a function of contralateral and ipsilateral time. C) Hemispheric delay. Histograms show the temporal delays between ipsilateral and contralateral times for highest positive interhemispheric correlations. Most top correlations occurred with a delay from contralateral to ipsilateral hemispheres. All data shown here are from the single-peripheral condition.

Figure S12. Consistency of information within versus across hemispheres, separately for each stimulus position.

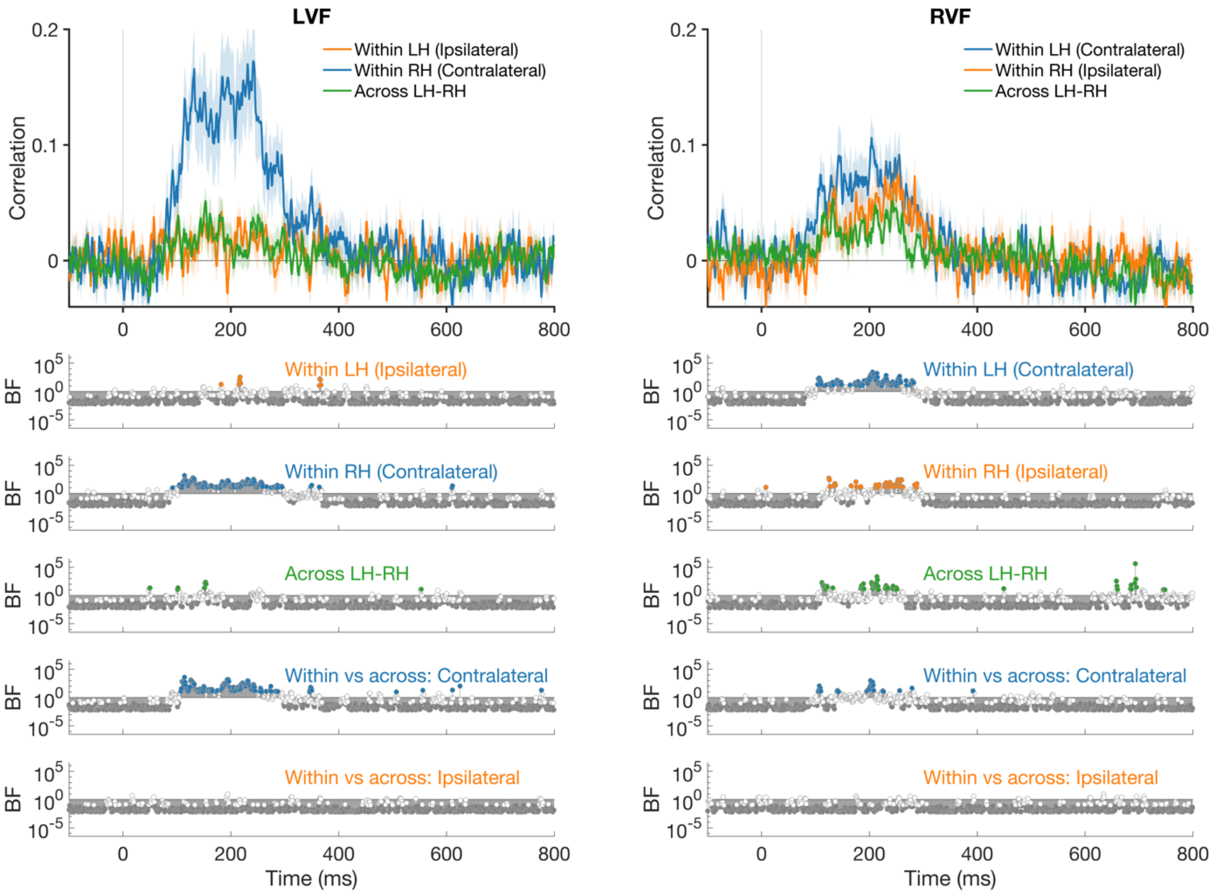

Note. Analyses additional to Figure 6 of the manuscript. Consistency of information is shown separately for left visual field stimuli (left plot) and right visual field stimuli (right plot). In both conditions, correlations between representational structure within and across hemispheres show unique information in the contralateral hemisphere above information that is shared with the ipsilateral hemisphere, but no unique information in the ipsilateral hemisphere. "Within" hemisphere consistency is calculated from a given hemisphere (contralateral/ipsilateral) using split-half Spearman correlation. "Across" hemisphere consistency is calculated as the correlation between the left and right hemispheres (as in Figure 5). Results are shown from the single-peripheral condition.

Figure S13. Neural-behaviour correlations for the stimulus similarity judgements.

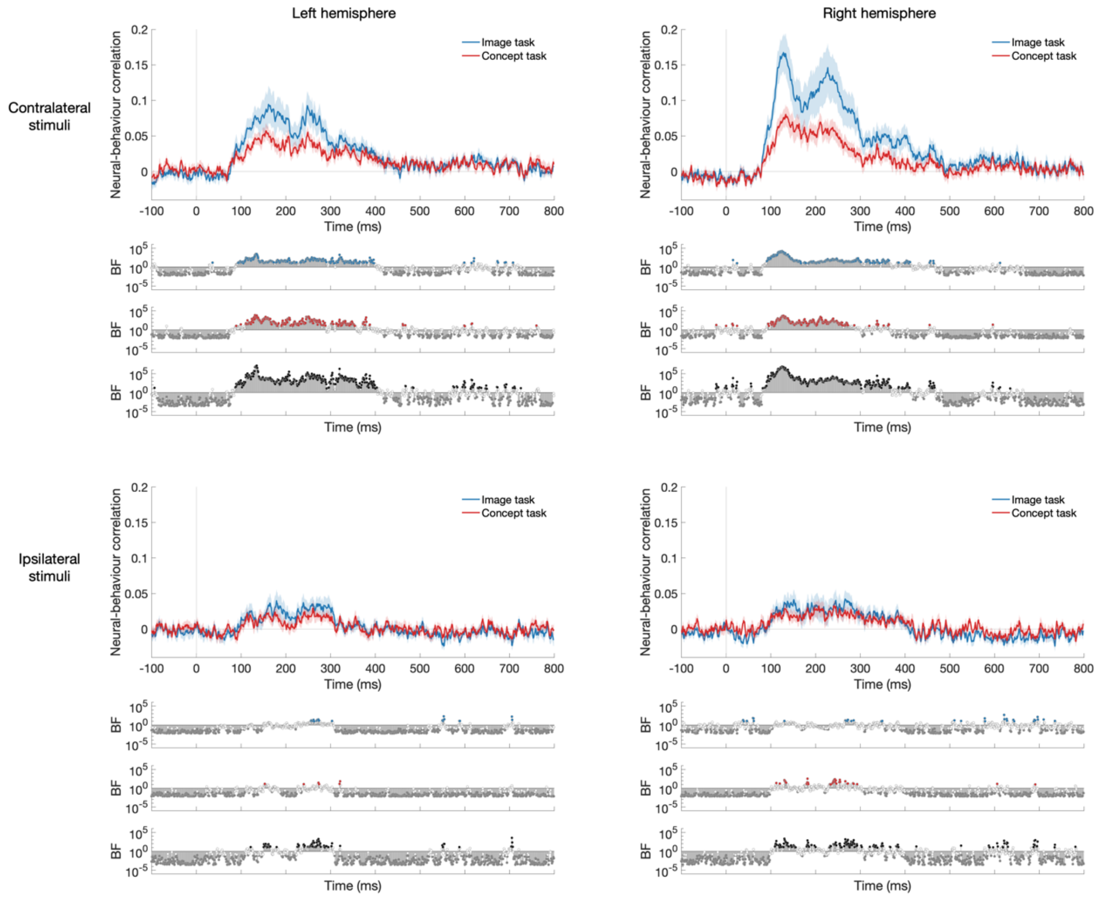

Note. Plots show correlations between neural stimulus dissimilarity per hemisphere and behavioural judgements on the image task and concept task, according to specific hemisphere and whether the stimuli were presented in the contralateral (top plots) or ipsilateral (bottom plots) visual field.

Figure S14. Decoding of peripheral images from right-handed participants.

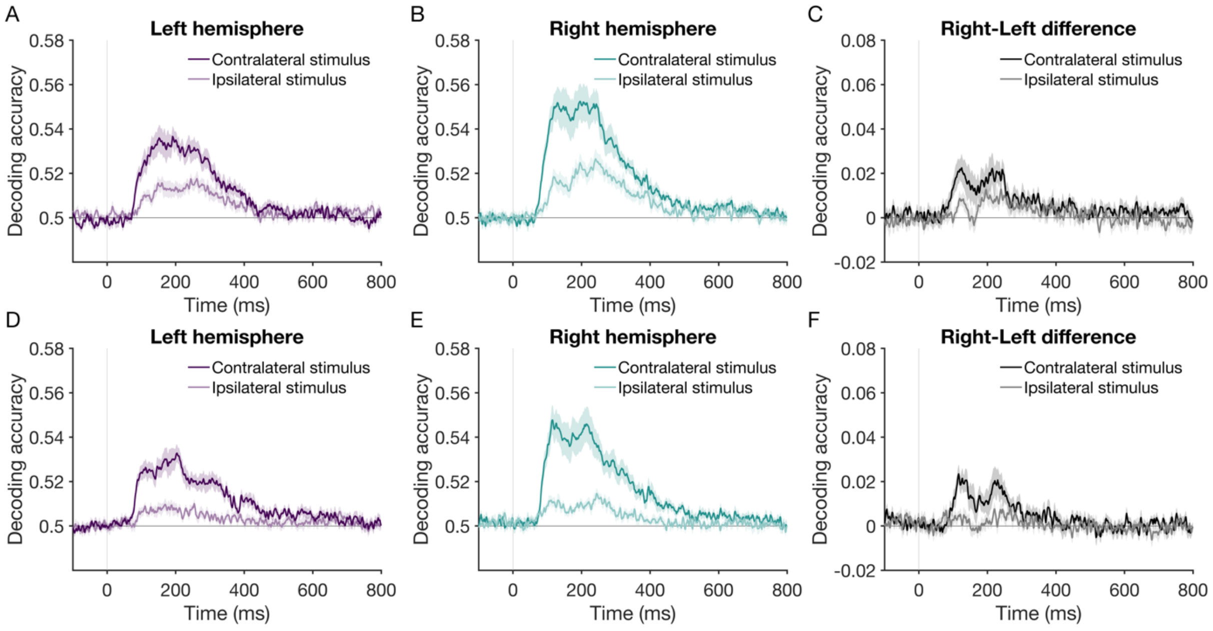

Note. Pairwise stimulus decoding for single-peripheral condition (A-C) and dual-peripheral condition (D-F), for the right handed individuals in the study (N = 17). The results are highly similar to the whole participant sample (Figures 2 and 3).

Tables

Table S1. Spearman correlations between behaviour on the odd-one-out tasks and stimulus models based on image characteristics or stimulus concept.

	'Image Task'	'Concept Task'	'Image Model'	'Concept Model'
'Image Task'		0.612*	0.811*	0.301*
'Concept Task'			0.279*	0.593*
'Image Model'				-0.062

Note. Behaviour on the image task and concept task were significantly correlated with both stimulus models. The image and concept models were not significantly correlated (p = .117). The image model was more strongly correlated with the image task than the concept task, and the concept model was more strongly correlated with the concept task. *p < .001.
